# High levels of interspecific gene flow in an endemic cichlid fish adaptive radiation from an extreme lake environment

**DOI:** 10.1111/mec.13247

**Published:** 2015-06-19

**Authors:** Antonia G. P. Ford, Kanchon K. Dasmahapatra, Lukas Rüber, Karim Gharbi, Timothee Cezard, Julia J. Day

**Affiliations:** ^1^ Department of GeneticsEvolution and EnvironmentUniversity College LondonLondonWC1E 6BTUK; ^2^ Department of BiologyUniversity of YorkYorkYO10 5DDUK; ^3^ Naturhistorisches Museum der Burgergemeinde BernBernastrasse 15Bern3005Switzerland; ^4^ Edinburgh GenomicsAshworth LaboratoriesThe University of EdinburghEdinburghEH9 3FLUK

**Keywords:** adaptive radiation, admixture, *Alcolapia*, cichlid, hybridization, Lake Magadi, Lake Natron, *Oreochromis*, RAD

## Abstract

Studying recent adaptive radiations in isolated insular systems avoids complicating causal events and thus may offer clearer insight into mechanisms generating biological diversity. Here, we investigate evolutionary relationships and genomic differentiation within the recent radiation of *Alcolapia* cichlid fish that exhibit extensive phenotypic diversification, and which are confined to the extreme soda lakes Magadi and Natron in East Africa. We generated an extensive RAD data set of 96 individuals from multiple sampling sites and found evidence for genetic admixture between species within Lake Natron, with the highest levels of admixture between sympatric populations of the most recently diverged species. Despite considerable environmental separation, populations within Lake Natron do not exhibit isolation by distance, indicating panmixia within the lake, although individuals within lineages clustered by population in phylogenomic analysis. Our results indicate exceptionally low genetic differentiation across the radiation despite considerable phenotypic trophic variation, supporting previous findings from smaller data sets; however, with the increased power of densely sampled SNPs, we identify genomic peaks of differentiation (*F*_ST_ outliers) between *Alcolapia* species. While evidence of ongoing gene flow and interspecies hybridization in certain populations suggests that *Alcolapia* species are incompletely reproductively isolated, the identification of outlier SNPs under diversifying selection indicates the radiation is undergoing adaptive divergence.

## Introduction

The study of adaptive radiation, the evolution of ecological diversity in rapidly multiplying lineages (Schluter 2000), is important in understanding the diversification of incipient species, as well as the genetic and ecological structure of species diversity (Hudson *et al*. 2011). Adaptive radiations from isolated insular systems, as opposed to more complex systems, offer clearer insight into fundamental evolutionary questions regarding the mechanisms generating biological diversity, and the role of ecological opportunity and sexual selection in the origin of species (e.g. Seehausen 2006, 2013; Gillespie 2013).

A major obstacle to studying recent radiations is insufficient genetic differentiation to define and characterize species relationships. However, the advent of high‐throughput sequencing has greatly facilitated the study of shallow divergence. Several recent studies have demonstrated the successful application of the reduced‐representation methodology of RAD (restriction‐site‐associated DNA) sequencing (Baird *et al*. 2008) to phylogenomic reconstruction (Rubin *et al*. 2012; Cariou *et al*. 2013; Eaton & Ree 2013; Wagner *et al*. 2013). Of these, Wagner *et al*. (2013) achieved exceptional phylogenetic resolution for the relatively young Lake Victoria cichlid fish adaptive radiation. These approaches, made possible by advances in sequencing technology, provide greater clarity on the basis for morphological species designation in cases where morphology does not match the molecular phylogeny (Keller *et al*. 2013). Furthermore, species delimitation is difficult in recent radiations using genealogical approaches of single‐gene or multigene alignments, and analyses indicate that population genomic approaches based on large sets of SNPs are more reliable in delimiting recently derived species (Shaffer & Thomson 2007).

**Figure 1:**
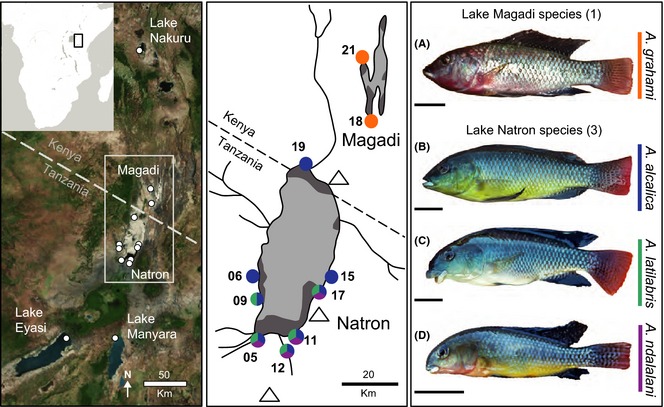
Map of soda lake sampling sites. Populations sampled in the present study are shown with white circles in the left‐hand panel. In the middle panel, sample site markers are coloured by species present at each site: *A. alcalica*, blue; *A. latilabris*, green; *A. ndalalani*, purple; and *A. grahami*, orange. Volcanoes are shown as open triangles; lake extent is shown in light grey, with open water lagoons designated by dark grey. The right‐hand panel shows the morphological diversity of the described *Alcolapia* species: (A) *A. grahami*; (B) *A. alcalica*; (C) *A. latilabris*; and (D) *A. ndalalani*. Scale bar: 10 mm. Colour bars to the right indicate colours used for respective species in all figures. Satellite imagery and mapping source: Esri, DigitalGlobe, GeoEye, Earthstar Geographics, CNES/Airbus DS, USDA, USGS, AEX, Getmapping, Aerogrid, IGN, IGP, swisstopo and the GIS User Community.

In this study, we examine species and population relationships within a very recent, small‐scale radiation of endemic cichlids, genus *Alcolapia*, which are the only fish occurring in the extreme environment of the East African soda lakes Natron (Tanzania) and Magadi (Kenya) (Fig. 1). Based on geological evidence, these cichlid species are thought to have diverged as recently as ~10 000 years ago (Williamson *et al*. 1993; Tichy & Seegers 1999). The soda lake system is similar to other recent, small‐scale freshwater fish radiations such as the neotropical crater lake cichlids (Barluenga & Meyer 2004; Elmer *et al*. 2010b, 2012), postglacial lake whitefish (Vonlanthen *et al*. 2009; Praebel *et al*. 2013) and three‐spined sticklebacks (Reusch *et al*. 2001; Aguirre *et al*. 2008) regarding its young geological age and highly restricted geographic area. These factors potentially make colonization inference more straightforward than in larger water bodies such as the African Great Lakes with older or less well‐defined geological histories and greater species diversity.

Both of the focal lakes are shallow endorheic basins, with Lake Natron having an average lake area of 398 km^2^ varying from 81 to 804 km^2^ (Tebbs *et al*. 2013), and Lake Magadi ranging from 75 to 108 km^2^ in dry to wet seasons (Jones *et al*. 1977; Vanden Bossche & Bernacsek 1990). The lakes are subject to substantial climatic effects, with a negative evaporative balance (Burrough & Thomas 2009). A thick layer of crystalline trona (sodium carbonate precipitate) covers most of the lakes' surfaces, forming a solid covering separating lagoons of permanent open water close to the shore (Kaufman *et al*. 1990). The area is volcanic with alkaline hydrothermal springs containing high levels of salts and precipitates feeding into the lagoons (Williamson *et al*. 1993). It is along these springs in which *Alcolapia* occur, although they also inhabit edges of the lagoons where the springwater meets the lake body (Narahara *et al*. 1996; Seegers & Tichy 1999). The lagoons are intermittently connected during heavy floods in the rainy season, which may allow migration of *Alcolapia* between populations usually restricted to isolated lagoons during the dry season (Seegers & Tichy 1999; Zaccara *et al*. 2014).

Currently, *Alcolapia* includes four described species (shown in Fig. 1), three of which are found within Lake Natron: *Alcolapia alcalica* (Hilgendorf 1905), *Alcolapia latilabris* (Seegers & Tichy 1999) and *Alcolapia ndalalani* (Seegers & Tichy 1999), occurring sympatrically at springs bordering the southern lagoon. *Alcolapia alcalica* is the only species with a lakewide distribution, also occurring in monospecific populations along the western and northern shores of Lake Natron (Fig. 1). Additionally, there are colour and trophic morphs found within certain Lake Natron *A. alcalica* populations (Seegers & Tichy 1999; Tichy & Seegers 1999; Seegers *et al*. 2001). The fourth species, *Alcolapia grahami* (Boulenger 1912), is restricted to Lake Magadi and satellite lake Little Magadi (Seegers & Tichy 1999), with a translocated population introduced to Lake Nakuru, Kenya, in the 1960s (Vareschi 1979). The four species exhibit extensive differentiation of trophic morphology, on which original descriptions were based (Seegers & Tichy 1999), with mouth morphology including terminal/retrognathous snout (*A. alcalica*), terminal/prognathous snout (*A. grahami*), inferior thick‐lipped (*A. latilabris*) and subterminal thin‐lipped (*A. ndalalani*) (Seegers & Tichy 1999). The species are also differentiated by breeding male coloration (Trewavas 1983; Seegers & Tichy 1999). Although no formal tests of species status and reproductive isolation have been conducted on *Alcolapia*, aquarium observations indicate preferential mating with conspecifics via female choice (Seegers *et al*. 2001). In mixed Lake Natron species tanks, male *A. alcalica* reportedly courted female heterospecifics without any successful spawning (Seegers *et al*. 2001), while hybrid *A. latilabris*/*A. ndalalani* were produced, but only when no male *A. ndalalani* were present and female *A. ndalalani* mated with dominant male *A. latilabris* (Seegers *et al*. 2001). Furthermore, the characteristic trophic morphology of the Natron species was maintained in laboratory populations over several generations (maintained up to *F*_6_), and did not differ in response to food type, indicating a genetic component rather than a plastic response to environment (Seegers & Tichy 1999; Seegers *et al*. 2001).

The conditions in the volcanic springs represent one of the most hostile environments to support fish life, including water temperatures of 30–42.8 °C, pH ~10.5, fluctuating dissolved oxygen levels of 0.08–6.46 mg/L and high salt concentrations (>20 ppt). Unique physiological adaptations to alkaline/saline and hypoxic conditions (mostly reported for *A. grahami*, with some limited study of *A. alcalica*) include the following: ureotelism (Randall *et al*. 1989), specialized gill structure for urea transport and excretion (Narahara *et al*. 1996; Walsh *et al*. 2001), high intracellular pH (Wood *et al*. 1994), a trifurcated oesophagus to prevent alkaline water diluting stomach acid (Bergman *et al*. 2003), and facultative air‐breathing via the air bladder (Maina 2000; Johannsson *et al*. 2014). Due to their uniqueness and morphological differentiation *Alcolapia*, originally a subgenus within *Oreochromis* (Thys van den Audenaerde 1968; Trewavas 1983), was elevated to genus (Seegers & Tichy 1999). However, this assignment renders *Oreochromis* paraphyletic as molecular phylogenies indicate that *Alcolapia* nests within *Oreochromis* (Seegers *et al*. 1999; Nagl *et al*. 2001; Schwarzer *et al*. 2009; Dunz & Schliewen 2013; Kavembe *et al*. 2013). Despite uncertainty over the generic status of *Alcolapia*, for this study we consider the constituent described *Alcolapia* species as valid taxonomic species, following the taxonomy of Eschmeyer (2015), and employing a phenotypic/cohesion species concept (e.g. Templeton 1989; Mallet 1995).

Lakes Natron and Magadi are situated in a basin that formed 1.7 Ma, and contained a single palaeolake Orolonga from ~700 ka (Eugster 1986) (Fig. S1, Supporting information). Based on geological evidence, the palaeolake exhibited lower salinity conditions than currently found (Eugster 1986). The separate lakes formed from the palaeolake during an arid event ~11 ka (Williamson *et al*. 1993), and the hypersaline and alkaline conditions of the current lakes arose ~7 ka (Roberts *et al*. 1993). Furthermore, fossils found in the high lake‐level beds surrounding Lake Magadi and ^14^C‐dated (9120 ± 170 years) are thought to be of an *Oreochromis* species exhibiting considerably larger body size than present‐day *Alcolapia* (Coe 1966; Trewavas 1983; Tichy & Seegers 1999). It therefore seems likely that *Alcolapia* adaptation to life in the extreme soda environment and subsequent diversification has occurred within a very short time frame. *Alcolapia* life history and the hostile environment are both conducive to rapid evolution. The short generation time recorded in these maternal mouth‐brooding cichlids (brooding period ~2 weeks, mating within seven weeks; Coe 1966; Trewavas 1983) is suggested to be a result of the increased metabolic rate required to inhabit elevated temperatures (McCormick *et al*. 2013). Such short generation times together with low effective population sizes allow for rapid allele fixation and drift mechanisms. Moreover, it has been suggested that not only did the lake contraction and increased salinity create a strong selection pressure when the two lakes separated and lagoons formed 7–10 000 years ago (Wilson *et al*. 2004), but also that the extreme environment of alkalinity, free radicals and exposure to UV light may promote new adaptations due to elevated mutation rates (Seegers *et al*. 1999; Pörtner *et al*. 2010).

Here, we aim to characterize *Alcolapia* evolutionary relationships through dense sampling employing a genomewide SNP approach. A genomic approach is well suited to this system as previous studies have been unable to resolve constituent species using mtDNA (control region 350–450 bp; cytb 420 bp) or microsatellite nuclear markers. These studies found shared haplotypes between the lakes and species (Seegers *et al*. 1999; Wilson *et al*. 2004; Zaccara *et al*. 2014), but also suggested some separation between the lakes, and an analysis of Lake Magadi (mtDNA 1913 bp; nuclear microsatellite loci) indicated structure between *A. grahami* populations (Kavembe *et al*. 2013). As well as phylogenomically testing species hypotheses, we test the population connectivity of *Alcolapia* within each of the lakes and examine the occurrence of interspecific gene flow within the radiation. Finally, we consider the distribution of differentiation heterogeneity across the genome and test for loci under selection between species.

## Methods

### Taxonomic sampling

Samples were collected in 2012 using hand, cast or seine nets (Table S1, Supporting information). Fish were euthanized using tricaine methanesulfonate (MS222) and preserved as voucher specimens in 80% ethanol, with genetic samples (fin clips) stored in 95% ethanol. Specimens were identified to species level according to the current taxonomic key (Seegers & Tichy 1999). Sampling locations are shown in Fig. 1. Straight‐line geographic distance between sampling locations was calculated using the Vincenty formula (Vincenty 1975) via GPS Visualizer (http://www.gpsvisualizer.com/calculators), and lake‐perimeter distances between sites were estimated using the GPS coordinates plotted in ArcGIS version 10 and summing over distances from intermediate sites.

### RAD library construction

A total of 96 individuals were sequenced for RAD‐tag generation comprising 88 *Alcolapia* specimens, and eight *Oreochromis amphimelas* samples from soda lakes Manyara and Eyasi (Table S1, Supporting information) selected as the outgroup (Trewavas 1983; Nagl *et al*. 2001). Genomic DNA was extracted from fin clips, using the DNeasy Blood and Tissue Kit (Qiagen). For degraded samples and those for which sufficient yield was not achieved using the Qiagen kits, DNA was extracted using a high‐salt chloroform/phenol protocol (methods in the Supplementary Information).

RAD library preparation, sequencing and preliminary bioinformatic processing were undertaken by Edinburgh Genomics (University of Edinburgh). Library preparation followed the protocol of Davey *et al*. (2013), using *SbfI* as the restriction enzyme. Samples were individually barcoded and multiplexed during preparation resulting in a total of six indexed RAD libraries. Libraries were sequenced using a 100‐bp paired‐end sequencing strategy on Illumina HiSeq 2000 (v3 chemistry). Libraries were initially sequenced across two lanes, but due to poor initial sequencing quality, each lane was resequenced once. One library showed a highly variable number of reads across samples and was therefore prepared again before sequencing in one‐third of a lane. Reads from all lanes were combined to maximize coverage.

### RAD SNP calling

RAD libraries were demultiplexed using the process_radtags function of STACKS v0.99993 (Catchen *et al*. 2011), and individual reads aligned to the *O. niloticus* reference genome Orenil1.1 (NCBI Assembly GCA_000188235.2, Brawand *et al*. 2014) using the Burrows Wheeler Aligner BWA‐backtrack function (Li & Durbin 2009). The resultant SAM files were converted to BAM files using SAMtools (Li *et al*. 2009) and duplicate reads marked for removal using PICARDtools (http://picard.sourceforge.net) to mitigate the effect of biased PCR amplification during library construction. BAM files were realigned around indels using the Genome Analysis Toolkit (GATK) 2.7–2 (McKenna *et al*. 2010). SNP genotyping was carried out using the GATK UnifiedGenotyper (DePristo *et al*. 2011) with default parameters and an emission confidence of 20, and run separately for *O. amphimelas*,* A. alcalica*,* A. grahami*,* A. latilabris* and *A. ndalalani,* with *O. niloticus* specified as the reference genome.

The resultant vcf files were filtered using custom perl scripts as used in Hoffman *et al*. (2014) at the following thresholds: SNP quality: 20; genotype quality: 20; mapping quality: 20; low coverage: five reads; and high coverage: 99.5 percentile of each sample's total coverage. Sites with missing data and those failing to pass quality thresholds were replaced with Ns in the matrix. Five individuals, each with fewer than 2.5 million bases passing the initial filtering step, were removed from the analysis. The final number of individuals passing this filtering step and included in downstream analyses was as follows: *A. alcalica*:* n* = 38; *A. latilabris*:* n* = 19; *A. ndalalani*:* n* = 15; *A. grahami*:* n* = 12; and *O. amphimelas*:* n* = 7. Full details and sequence quality measures by individual sample are given in Table S1 (Supporting information). The filtered species vcf files were merged to form a single alignment file. Further filtering included imposition of maximum levels of missing data and a minimum allele frequency threshold; these parameters differed by analysis, so the specific values for each analysis are described below and in Table S2 (Supporting information).

For reads that did not align to the reference genome, unmapped reads were extracted, read1 was clustered using STACKS with a minimum threshold for clustering of 70 of 96 individuals, and corresponding read2 assembled *de novo* using IDBA‐UD (Peng *et al*. 2012). A consensus sequence was then generated from the resultant reads, and any reads not mapping back to the consensus were discarded. All downstream processing and SNP calling for the unmapped reads followed that described above for the reference‐aligned reads, using the consensus sequence as the pseudo‐reference from which to make genotype calls in the GATK.

### Estimation of the extent of linkage disequilibrium

As several downstream analyses required the use of unlinked SNPs, and linkage disequilibrium (LD) has not previously been investigated in *Alcolapia*, we estimated LD for each species using the R package snpStats (Clayton & Leung 2007), with R scripts modified from Martin *et al*. (2013), using the reference‐aligned data set. We estimated LD between pairs of SNPs on each linkage group and averaged the correlation coefficient *r* ^2^ of all pairs within specified distance bins. We estimated background LD by calculating *r* ^2^ between all pairs of SNPs on different linkage groups. We did not include in our calculations any scaffolds that were not assigned to specific linkage groups of the reference genome.

### Phylogenomic inference

Maximum‐likelihood (ML) phylogenetic inference was conducted using RAxML (Stamatakis 2014) implementing a rapid bootstrap search on all data sets (Table S2, Supporting information), with *O. niloticus* (for mapped reads) or *O. amphimelas* (for *de novo*‐assembled reads) specified as the outgroup for 100 bootstrap replicates. Most analyses were performed within the CIPRES Science Gateway V. 3.3 (Miller *et al*. 2010) using the RAxML‐HPC2 version on XSEDE, with the default models using GTRCAT for the bootstrapping phase and GTRGAMMA for the final tree inference. We used a reduced‐taxon data set for ML analysis of the full alignment (including invariant sites), and selected the sample(s) from each population with the highest sequencing quality, for a total of 25 taxa. This full‐alignment data set was analysed using the SSE PTHREADS version of RAxML 8. For SNP‐only data sets (i.e. alignments including no invariant sites), we ran the RAxML GTRGAMMA model with and without the correction for ascertainment bias (ASC) that may be more appropriate for SNP alignments containing no constant sites (RAxML 8 Manual); however, as the ASC_model runs only on variant sites and does not consider ambiguous bases variable if the base could be the same as determined bases at that site, this necessitated using a different data set (excluding ambiguous bases). Given the difficulty in estimating ingroup relationships within this data set (see Results), we also used a Neighbour‐Net algorithm (Bryant 2004) based on uncorrected p‐distances implemented in SplitsTree 4.13.1 (Huson & Bryant 2006) and drawn using the equal‐angle algorithm.

A species tree was estimated using the Bayesian software program snapp v 1.1.4, (Bryant *et al*. 2012) as an add‐on package to BEAST v 2.1.3 (Bouckaert *et al*. 2014). Owing to the prohibitive increase in computational requirements with increasing taxa and individual number, we used a reduced data set. We predefined population membership based on taxonomic species by sampling site, and included sites where Lake Natron species occurred sympatrically and full sample sizes were available (sites 05 and 11), sites exhibiting *A. alcalica* morphs (site 15), the northernmost Natron sampling site (site 19) and only Lake Magadi *A. grahami* sites (18 and 21). The data set comprised all biallelic SNPs across 44 *Alcolapia* samples (four samples for each of 11 populations) and four *O. amphimelas* samples, with a minimum distance between SNPs of 500 kb on each linkage group (to ensure SNPs were unlinked), and we removed any sites for which data were not available for all samples, leaving a total of 1266 SNPs. Backward and forward (*u* and *v*) mutation rates were estimated from the data using equation 8.4.1 from (Drummond & Bouckaert 2014) and fixed at their initial values of *u *=* *1.3420 and *v *=* *0.7969. We used a gamma prior with parameters to account for small population sizes (α = 2, β = 2000, with θ = 0.001), and each analysis was run for 7 million generations, discarding the first 10% as burn‐in. Runs were checked for convergence using tracer v 1.5 software (Rambaut & Drummond 2007), ensuring that each reached an effective sample size (ESS) > 200. Resultant tree sets were visualized using DensiTree (Bouckaert 2010).

### Population genomic analyses

Population clustering of *Alcolapia* populations was assessed using STRUCTURE v 2.3.4 (Pritchard *et al*. 2000). As the underlying clustering algorithm of STRUCTURE assumes markers are unlinked loci, we used biallelic SNPs and imposed a minimum distance of 500 kb between SNPs on the same linkage group, resulting in a data set of 2297 SNPs across the 84 *Alcolapia* samples (Table S2, Supporting information). For comparison, we also ran these analyses on the full data set without accounting for linkage disequilibrium. Given the very recent divergence of *Alcolapia* species, we also ran the analysis using the LOCPRIOR model (Hubisz *et al*. 2009) using taxonomic species as a prior, which can provide more accurate inference of population structure when the signal is too weak for standard STRUCTURE models to detect. For species priors, we used the four described species, and additionally included a 5th category for two samples that were originally identified as *A. alcalica*, but after further inspection were reclassified as *A*. aff. *ndalalani* (see Results).

The allele frequency parameter (*λ*) was estimated using an initial run of *K* = 1 with 50 000 burn‐in and 100 000 further iterations, giving a value of λ = 0.5252. This value was set in subsequent runs of 5 iterations at each value *K* = 1–12 with no prior population information, and 50 000 burn‐in/100 000 further iterations. Analyses were run with all different model parameters independently (total of four separate analyses: admixture model/allele frequencies correlated (default settings); admixture model/independent allele frequencies; no admixture/allele frequencies correlated; no admixture/allele frequencies independent). STRUCTURE output was compiled and averaged using Structure Harvester (Earl & vonHoldt 2011) to conduct the Evanno method (Evanno *et al*. 2005), and run permutations were clustered using CLUMPP v 1.1.2 (Jakobsson & Rosenberg 2007). Finally, clustered output was visualized using Distruct v 1.1 (Rosenberg 2004). Additional STRUCTURE runs on data subsets (including only sympatric Lake Natron populations, and including only *A. alcalica* populations) are described in the Supplementary Information.

### Pairwise comparisons

Uncorrected pairwise p‐distances between samples were calculated in the package ape using r v 2.15.2 (R Core Team 2012). Calculations of pairwise *F*_ST_ to test genomic differentiation between populations were conducted in the EggLib Python module (De Mita & Siol 2012). Whole‐data set *F*_ST_ values were estimated by averaging over nonoverlapping windows of 100 kb, which has been shown to provide accurate estimates for small sample numbers (Nadeau *et al*. 2012). Any windows returning negative values for *F*_ST_ were removed before averaging. We also calculated *F*_ST_ in Arlequin 3.1.5.2 (Excoffier *et al*. 2005), accounted for differences in sample size between populations, and tested significance using 10 100 permutations.

As recently diverged species are likely to continue to exchange genes through interspecific hybridization (e.g. Nosil *et al*. 2009b), we examined the extent of ongoing gene flow between species using the *f*_*4*_ four‐population test for admixture (Reich *et al*. 2009, 2012), which is based on the fact that genetic drift should be uncorrelated in unadmixed populations. We used the *f*_4_ test rather than tests of phylogenetic discordance (e.g. ABBA‐BABA tests; Durand *et al*. 2011), which may be confounded by the presence of gene flow between sympatric taxa. The test was conducted between each of the Natron species at three sympatric sites with varying geographic distance (sites 05, 12, 17). We calculated the *f*_*4*_ statistic mean and variance with a block jackknifing approach (block size of 500 kb, as identified by LD estimates) using modified python scripts adapted from Martin *et al*. (2013; Dryad Digital Repository. doi: 10.5061/dryad.dk712). The *F*_ST_ and *f*_*4*_ statistics were calculated using only reads that aligned to assigned linkage groups within the reference genome. Following phylogenomic analysis and an unexpected placement within the resulting phylogeny (see Results section), two samples were found to represent intermediate morphology between species and were excluded from population comparisons for *F*_ST_ and the *f*_4_ test.

### Genomic scans and *F*_ST_ outlier loci

To investigate differentiation across the genome, we calculated relative (*F*_ST_) and absolute (D_XY_) sequence divergence between species in sliding‐window analyses conducted in EggLib (De Mita & Siol 2012). Pairwise comparisons were performed between all species in Lake Natron, between *A. alcalica*/*A. grahami* and also *A. alcalica*/*O. amphimelas*. Populations were predefined and individuals selected to ensure even numbers in each comparison. Numbers were constrained to eight individuals (*Alcolapia*) as the maximum sample number of *A. grahami* from Lake Magadi, or seven samples (*O. amphimelas*), as the maximum sample number from lakes Manyara and Eyasi. Where more than the predefined number of individuals were available, selection was made based on geographic sampling and sequence quality of the RAD data. Only individuals from southern populations (sites 05 and 11) were included for *A. alcalica*. Analyses were run on the entire set of *Alcolapia* filtered biallelic SNPs (91 individuals; 22.2 Mb; data set C) with a window size of 1 Mb, a slide length of 100 kb, included only windows with a minimum of 10 000 sites, and excluded any unplaced scaffolds.

To assess whether the top 1% and 5% *F*_ST_ outliers were distributed nonrandomly across the genome, we also calculated *F*_ST_ in nonoverlapping windows (to preclude nonindependence of windows), using a window size of 100 kb, a slide length of 100 kb and a minimum of 1000 sites. We tested nonrandom distribution of outlier windows using 10 000 permutations, comparing the closest interpeak distances between observed and permuted data sets, and employing a nearest neighbour index (NNI) as an indicator of the level of clustering in the observed data (Clark & Evans 1954). We calculated a modified NNI ratio, which used the mean of our permuted data as the ratio denominator (rather than the standard random‐distribution denominator of points/distance), to avoid the assumption of a purely linear genome, and for which we only calculated distances within linkage groups. We used a Z‐statistic to test whether the modified NNI was significantly different from the mean random distribution (Clark & Evans 1954; Hammond & McCullagh 1978). We used bin numbers for the distance calculations (for each nonoverlapping window) rather than taking a mid‐point chromosomal bp location, although this had no effect on the significance of the results (tested in 50% of comparisons). All permutations and significance testing were conducted in R 3.1.2.

We also estimated the number of loci under selection by looking for *F*_ST_ outlier loci implementing a Bayesian approach in bayescan 2.1 (Foll & Gaggiotti 2008) between the same populations as for the sliding‐window analyses. For these analyses, a minimum allele frequency of 10% was imposed, with a missing data threshold at each site of 25% across all individuals in each comparison. Input files were formatted using PGDSpider 2.0.8.0 (Lischer & Excoffier 2012), and we set prior odds for the neutral model at 10, using default parameters for the MCMC analysis. We analysed these results to identify outlier loci at false discovery rates of FDR = 0.10 and FDR = 0.05.

### Isolation by distance

To investigate the hypothesis of panmixia within the lakes, we used Mantel tests to test for covariation of genetic distance with geographic distance between populations. Mantel tests were conducted using the ade4 package (Dray & Dufour 2007) to test matrix covariation in R 2.15.1 for population pairwise *F*_ST_ comparisons vs. geographic distance between sampling sites.

## Results

### Generation of a genomewide SNP data set using RAD Sequencing

A total of 83.6 Gb of sequence was produced, of which 89% successfully mapped to the *O. niloticus* reference genome in the alignment stage. Mapping, duplication and filtering statistics are provided in the Supplementary Information in Table S1 (Supporting information). The final data sets used in different analyses for phylogenomic inference and population genomic analysis are detailed in Table S2 (Supporting information).

### Linkage disequilibrium

We calculated *r* ^2^ for pairs of SNPs on the same linkage group and plotted against distance between SNPs. LD decreased with distance (Fig. S2, Supporting information) and reached background level (mean LD between SNPs on different linkage groups) at 100–500 kb. This distance was smaller in *A. alcalica* and *A. grahami* than in *A. latilabris* and *A. ndalalani*, suggesting larger population sizes in these species. For all downstream analyses that required unlinked SNPs, we imposed a minimum distance between SNPs of 500 kb.

### Phylogenomic inference

**Figure 2:**
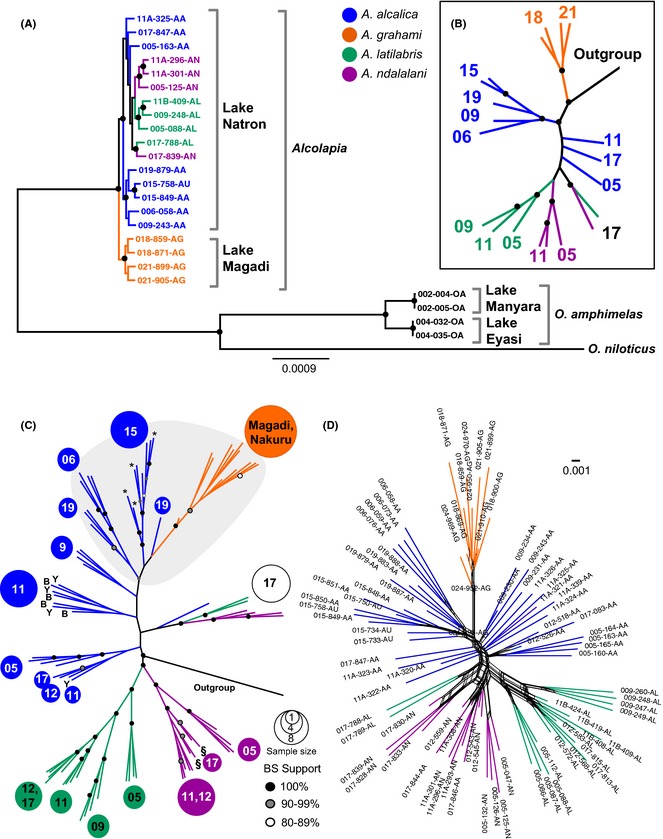
Phylogenomic analysis of RAD sequences aligned to the *O. niloticus* reference genome. (A) Maximum‐likelihood phylogeny (RAxML) for the reduced‐taxon data set (*n* = 25), full quality‐filtered alignment (data set B; 26 million bp); (B) radial tree layout for tree in panel A; (C) ML phylogeny of the full taxon data set (*n* = 92) alignment of variable sites only (data set D; 544 916 SNPs); (D) phylogenetic network (Neighbour‐Net) of ingroup taxa (data set M; 84 samples, 246 336 SNPS). (B–C) Numbers at tips indicate the sampling location (population) of individuals in each clade; branch length to outgroup has been truncated for clarity. (C) Northern Lake Natron sites and Magadi/Nakuru sites are shaded in grey. * indicates *A. alcalica* upturned‐mouth morph individuals from site 15. § indicates possible hybrids that displayed intermediate morphology between *A. alcalica* and *A. ndalalani* from site 17. B and Y at tips indicate, respectively, blue or yellow *A. alcalica* morphs found at site 11.

The ML phylogeny of the full mapped‐read alignment reduced‐taxa data set (data set B; 26 million bp; *n* = 25) provides maximum support of a clade composed of *A. grahami* individuals as sister to the clade comprising Lake Natron *Alcolapia* (Fig. 2A, B). However, there is weak support for the monophyly of the Lake Natron *Alcolapia* species, with *A. alcalica* from the northern populations sister to all species from the sympatric southern populations (i.e. *A. alcalica*,* A. ndalalani*,* A. latilabris*). Short branch lengths within the ingroup relative to the outgroup indicate very low genomic differentiation. In contrast, there is maximum support for the separation of *O. amphimelas* (outgroup) populations between Lake Eyasi and Lake Manyara, and branch lengths between these populations are considerably longer than those across the entire *Alcolapia* radiation.

Considering the *Alcolapia* populations in more detail, we conducted ML analysis on the full taxon data set, but included only SNPs in the analysis (data sets D & E; Figs 2C and S3, Supporting information). The SNP‐only phylogenies indicate that the Lake Natron clade is nonmonophyletic, but support for this relationship is weak. The two species restricted to the southern lagoons, *A. latilabris* and *A. ndalalani,* form well‐supported sister clades to the exclusion of individuals from site 17, while the ubiquitous Lake Natron species *A. alcalica* comprises two separate clades separated by sampling locality of northern and southern sites.

Notably, most taxa from site 17 comprise a clade, rather than clustering by species, and are sister to the northern *A. alcalica* clade. The genomic signal from individuals at site 17 does not reflect existing species designations. While all four *A. ndalalani*, two *A. latilabris* samples and a single *A. alcalica* specimen form a separate, well‐supported clade, other site 17 individuals occur elsewhere within the tree and two *A. alcalica* samples nest within *A. ndalalani*. We reexamined voucher material for all site 17 specimens and were confident with original morphology‐based species identification for all samples except the two *A. alcalica* individuals nesting within *A. ndalalani* (017‐844‐AA and 017‐846‐AA) that exhibited an intermediate form, and we thereafter designated them *A*. aff. *ndalalani*.

The ML tree also generally exhibits populations (sampling sites) that cluster together within these clades. Given the uncertainty of some branch placements and low bootstrap support for *A. alcalica* nodes, we also visualized this data set as a phylogenetic network based on the *Alcolapia* samples only (246 366 SNPs, data set M; Fig. 2D). The network again highlights the close relationships of all Lake Natron species, and while *A. grahami* nests with *A. alcalica*, this species is well separated from the Natron species (Fig. 2D). Across all the phylogeny and network analyses, there was no discernible genomic differentiation between intraspecific morphs.

Maximum‐likelihood trees generated from the *de novo* assembly data sets (Table S2, Supporting information; data sets G, H and J) did not resolve clades within *Alcolapia*, and individuals did not cluster by site or species. Given the lack of resolution for almost all nodes, we present these phylogenies as majority rule (50%) consensus trees, and almost the entire *Alcolapia* forms a polytomy (Fig. S4 (A–C), Supporting information). However, the 100% bootstrap support for *O. amphimelas* nodes was maintained – both for the node separating *O. amphimelas* from *Alcolapia*, and the node separating *O. amphimelas* specimens between lakes Manyara and Eyasi. When the *de novo* data sets were combined with the mapped‐read data set (data sets K and L), ML analysis exhibited similar topology to the alignment of mapped reads alone and grouped by species, but with lower bootstrap support (Fig. S4 D and E, Supporting information).

Similar to ML analysis of the full alignment (data set B), the SNAPP species tree (Fig. S5, Supporting information) also places *A. grahami* as sister to a clade composed of all Lake Natron terminals, but reveals a deeper divergence between this taxon and those from Lake Natron. Furthermore, the species analysis demonstrated long branch lengths to the outgroup taxa, but very close relationships within *Alcolapia* species from southern Natron populations.

### Population clustering and admixture

**Figure 3:**
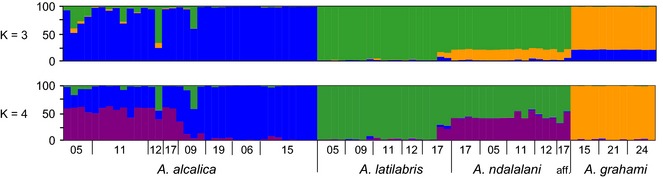
STRUCTURE analysis of *Alcolapia* populations. Analysis of the data set accounting for linkage disequilibrium, imposing a minimum distance of 500 kb between SNPs (data set N; 84 samples; 2297 unlinked biallelic SNPs), using the admixture and correlated allele frequency models with STRUCTURE. Clusters are visualized for the most likely *K* values (*K* = 3–4). Results for alternative models and the full SNP alignment are provided in the Supplementary Information (Figs S5–S7, Supporting information).

STRUCTURE analysis of *Alcolapia* unlinked SNPs (data set N) gave the highest likelihood scores for the admixture and correlated allele frequencies models. Using these models, lnP(K) gave an optimum of *K* = 4 and the Evanno method exhibited a modest peak at *K* = 3 (both K values visualized in Fig. 3). Running the analysis with the LOCPRIOR model and species prior information gave a clear optima of *K* = 3, as did running the analysis across the full data set (not accounting for LD; data set M; Fig. S6, Supporting information). The cluster membership at *K* = 3 and *K* = 4 reflects the differentiation observed in the ML tree, with shared cluster membership between species in the sympatric southern Lake Natron populations, but with the allopatric northern populations of *A. alcalica* showing strong probability of membership to a single cluster. Furthermore, *A. grahami* is assigned to a distinctly separate cluster from all other individuals, with no mixing.

As the STRUCTURE results indicated high levels of admixture between species, we conducted four‐population tests specifically designed to test admixture. The *f*_*4*_ population test comparisons were all significant, indicating admixture between all species pair combinations: *A. alcalica*–*A. ndalalani*,* A. alcalica*–*A. latilabris* and *A. latilabris*–*A. ndalalani* (Table 1). Furthermore, the test *Z*‐score for the comparison of *A. latilabris* and *A. ndalalani* at site 17 was substantially higher than that for other comparisons, suggesting increased gene flow between species at this site.

**Table 1:** Four‐population test for recent gene flow

A	B	C	D	*f*_4_ ± SEM	*Z*‐score	*P*‐value
05_Aa	12_Aa	05_An	12_An	0.011 ± 0.002	6.369	1.9 × 10^−10^
05_Aa	12_Aa	05_Al	12_Al	0.012 ± 0.002	6.830	8.5 × 10^−12^
05_An	12_An	05_Al	12_Al	0.012 ± 0.002	7.400	1.4 × 10^−13^
05_Aa	17_Aa	05_An	17_An	0.010 ± 0.001	9.782	1.3 × 10^−22^
05_Aa	17_Aa	05_Al	17_Al	0.010 ± 0.001	10.672	1.4 × 10^−26^
05_An	17_An	05_Al	17_Al	0.021 ± 0.001	21.522	9.8 × 10^−103^

The *f*_4_ statistical test between 2 pairs of populations (A,B; C,D) – A significant *Z*‐score indicates gene flow, with positive values implying flow between populations A–C and/or B–D. Aa: *A. alcalica*; Al: *A. latilabris*; and An: *A. ndalalani*. The number in each population name refers to the sampling location as numbered in Fig. 1.

Interpopulation *F*_ST_ values exhibited a similar pattern when calculated from the entire data set using EggLib or when using a reduced data set (maximum 10% missing data) in Arlequin, so we present only the latter set of results here, as Arlequin accounts for differences in sample size. Pairwise population *F*_ST_ values revealed higher levels of differentiation between more distant populations and between described species (Table S3, Supporting information). As expected, *A. grahami* populations exhibited the highest *F*_ST_ values when compared to Lake Natron populations, indicating population structuring between the two lakes, with lower pairwise *F*_ST_ values between *A. grahami* and the most northern *A. alcalica* population (site 19). Individuals from site 17 in Lake Natron exhibited the lowest interpopulation and interspecific values, which correlates with relationships as indicated by the ML tree and suggests very recent or contemporary hybridization occurring at this site. The range of intraspecific *F*_ST_ values between populations was similar across Lake Natron species (*A. alcalica*: 0.001–0.113; *A. latilabris*: 0.030–0.146; *A. ndalalani*: 0.000–0.143), but substantially lower in Lake Magadi *(A. grahami*: 0.000–0.014). However, after correcting for multiple tests (Bonferroni correction), none of these population comparisons was significant.

### Outlier loci

**Figure 4:**
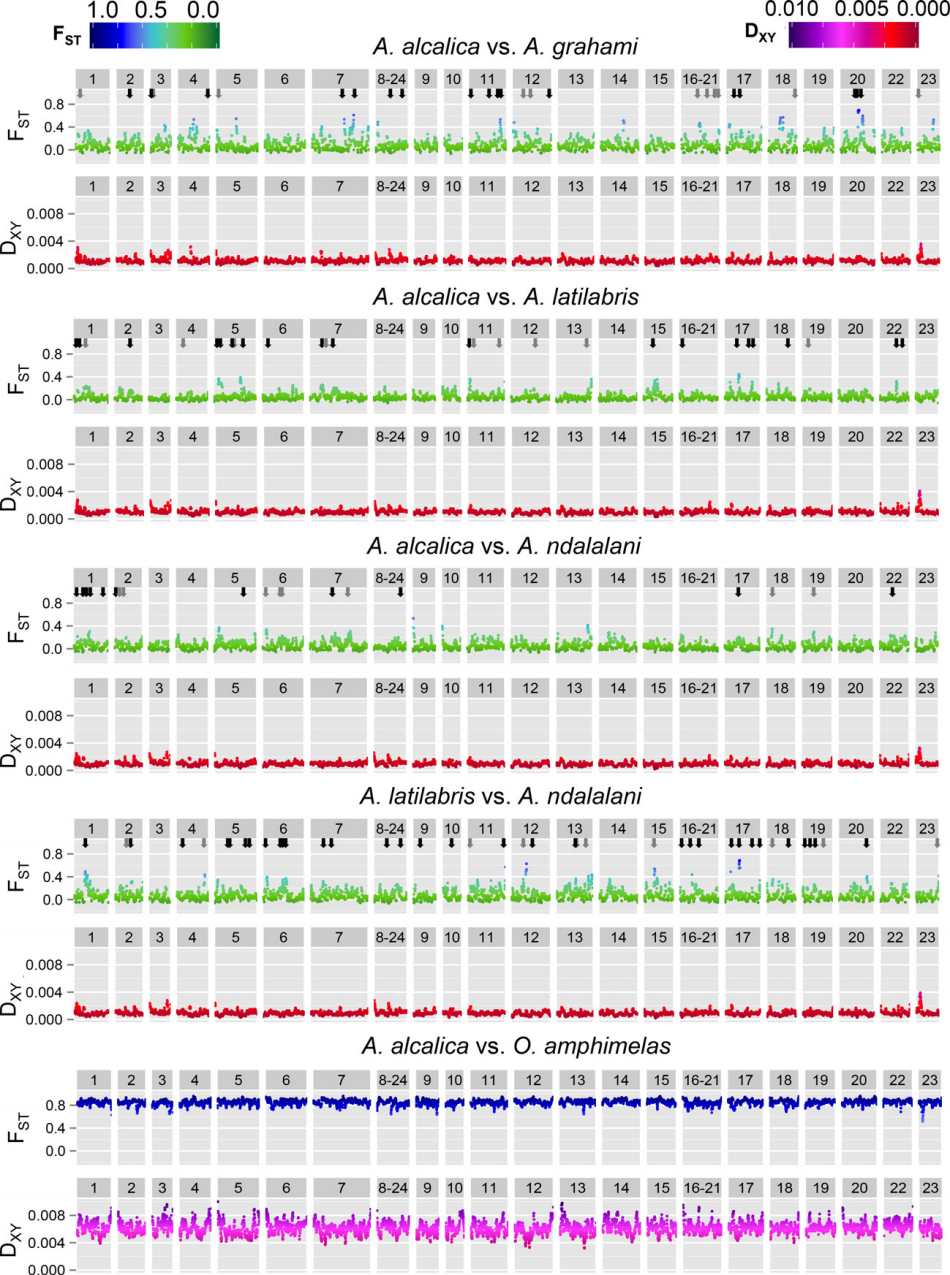
Sliding‐window analysis of relative (*F*_ST_) and absolute (D_XY_) divergence for pairwise species comparisons. Eight samples per species were used for each comparison except for the final comparison, which was based on seven samples per species. Results are plotted by linkage group of the reference genome (*O. niloticus*) as indicated by numbers in the upper grey bar of each plot. Window size is 1 Mb with a slide of 100 kb. Approximate genome position of *F*_ST_ outliers identified by bayescan is indicated by arrows for FDR = 0.05 (black) and FDR = 0.10 (grey).

Sliding‐window analyses of *F*_ST_ indicated heterogeneous differentiation across the genome in the *Alcolapia* comparisons, with several peaks of divergence in each pairwise comparison against a background of low divergence (Fig. 4). This is in contrast to the *A. alcalica*/*O. amphimelas* comparison, which exhibited uniformly high values of *F*_ST_ across the genome. The D_XY_ analyses showed less substantial variation across the genome, and fewer peaks of high diversity, but the highest peak was found in all within‐*Alcolapia* comparisons on linkage group 23 (Fig. 4), although this peak was not identified by the bayescan analysis. Plotting the frequency distribution of the sliding windows (Fig. S9, Supporting information) exhibited a right‐skewed pattern for within‐*Alcolapia* comparisons with a majority of windows showing low differentiation, but a small number showing comparatively high *F*_ST_ values. Conversely, the *Alcolapia*‐outgroup comparison showed a left‐skewed distribution, with most comparisons showing high levels of differentiation and only a few regions of low differentiation. We also calculated *F*_ST_ values for nonoverlapping windows, and plotting the top 1% and 5% *F*_ST_ windows in the genomewide analysis indicated heterogeneous distribution across the genome (Fig. S10, Supporting information). The range of *F*_ST_ values covered by the top 5% of values was considerably larger within the *Alcolapia* comparisons (*F*_ST_ = 0.2–0.8) than in the *Alcolapia*‐outgroup comparison (*F*_ST_ = 0.98–1.00). The distribution of 1% outliers was significantly nonrandom only in the *A. alcalica* vs. *A. latilabris* comparison (significant by permutation testing and *Z*‐statistic of the NNI ratio); however, all comparisons exhibited NNI < 1, indicating tendency to clustering rather than dispersion.

bayescan analysis identified several *F*_ST_ outliers in each of the *Alcolapia* comparisons (Table 2), but no outliers in the *A. alcalica*/*O. amphimelas* comparison (even at the relaxed false discovery rate FDR = 0.1). The details of all the loci identified as outliers for *Alcolapia* comparisons are given in Tables S4–S7 (Supporting information), and the corresponding gene annotations for loci identified in more than one species comparison in Table S8 (Supporting information). All of the outliers indicated diversifying selection (alpha > 0) rather than balancing selection, and the majority of outliers had log_10_ Bayes factor scores >0.5 (considered substantial on Jeffrey's scale of evidence; Foll 2012). For each comparison, these were as follows: *A. alcalica*/*A. grahami*: 86%; *A. alcalica*/*A. latilabris*: 87%; *A. alcalica*/*A. ndalalani*: 100%; and *A. latilabris*/*A. ndalalani*: 96%.

**Table 2:** *F*_ST_ outlier analyses

Comparison	All SNPs		Outliers (FDR = 0.05)		Outliers (FDR = 0.10)	
	*N*	*F*_ST_ range	*n* (%)	*F*_ST_ range	*n* (%)	*F*_ST_ range
*A. alcalica* vs. *A. grahami*	23 264	0.01–0.49	31 (0.13)	0.32–0.49	45 (0.19)	0.27–0.49
*A. alcalica* vs. *A. latilabris*	30 841	0.04–0.35	39 (0.13)	0.16–0.35	55 (0.18)	0.15–0.35
*A. alcalica* vs. *A. ndalalani*	28 026	0.04–0.28	14 (0.05)	0.18–0.28	27 (0.10)	0.16–0.28
*A. latilabris* vs. *A. ndalalani*	22 946	0.05–0.42	43 (0.19)	0.22–0.42	75 (0.33)	0.18–0.42
*A. alcalica* vs. *O. amphimelas*	82 474	0.68–0.85	0	–	0	–

*F*_ST_ outliers were identified using bayescan (Foll & Gaggiotti 2008) with false discovery rates (FDR) of 0.05 and 0.10. All outliers identified in each comparison exhibited alpha >0, indicative of diversifying selection. Full details of outliers are given in Tables S4–S7 (Supporting information).

### Phylogenomic covariation with geography

To investigate whether each species exhibits panmixia within the respective lakes, we tested isolation by distance by comparing pairwise population *F*_ST_ comparisons within species to geographic distance between sampling sites. None of the population comparisons exhibited significant covariation with geographic distance (Table S9, Supporting information), indicating that genomic divergence between populations is not correlated with distance.

### *Alcolapia* phylogenomic differentiation

Across the whole data set, there was very low phylogenomic differentiation, with a mean interindividual uncorrected p‐distance for the *Alcolapia* of 0.020%, which was the same as for that within Lake Natron alone (0.019%), and mean interspecific distances only marginally larger: 0.020–0.026% (Fig. S11, Supporting information). As we focused our analyses on reads aligned to the *O. niloticus* reference genome, we also *de novo*‐assembled reads that did not align to the genome in case these reads represented regions of the genome that had substantially diverged from *O. niloticus*. However, these reads did not appear any more divergent from the outgroup *O. amphimelas* species than aligned reads based on genetic distance (uncorrected p‐distance; Fig. S11, Supporting information).

## Discussion

Despite clear morphological differences and unique physiological adaptations in soda lake cichlids, previous genetic work has been unable to resolve relationships within the *Alcolapia* radiation. Here, we present an extensive genomic data set containing dense sampling of the entire *Alcolapia* and address existing species hypotheses and phylogeny of the entire radiation.

### *Alcolapia* species relationships

Consistent with described species, the ML tree (Fig. 2A) achieved maximum support for Lake Magadi *A. grahami*, as well as the geographically restricted clades comprising *A. latilabris* and *A. ndalalani*, excluding the anomalous sampling site 17 clade. However, we unexpectedly find the geographically widely distributed *A. alcalica* to be comprised of two clades – clustering by northern and southern localities. This finding is likely to result from geographic isolation of certain *A. alcalica* populations (particularly between the northern and southern lagoons) coupled with gene flow between the southern *A. alcalica* and the other sympatric species. The widespread occurrence of *A. alcalica* across Lake Natron (Fig. 1) means that several populations are separated by extensive stretches of trona, and there is currently not continuous open water between the northern and southern lagoons. The Neighbour‐Net network (Fig. 2D) is congruent with the phylogenetic analyses, showing species‐level clustering of *A. grahami*,* A. latilabris* and *A. ndalalani* (excluding site 17 individuals), with higher levels of reticulation between site 17 individuals and the *A. latilabris*/*A. ndalalani* clusters than over the rest of the network. *Alcolapia alcalica* did not form a clade, but clustered by population from the centre of the network, while *A. grahami* appeared the most distinct taxon and had the least reticulation with other groups. The SNAPP analysis (Fig. S5, Supporting information) produced a species tree topology largely congruent with ML analysis and confidently resolved *A. grahami* as sister to all the Lake Natron species, with less certainty of Lake Natron species relationships.

A potential difficulty in the interpretation of our phylogenomic analysis is the placement of the root in the different tree topologies. As the outgroup (*O. amphimelas*) is comparatively distant to the ingroup that has diverged rapidly, it is difficult to place the root accurately, which in turn can influence the ingroup topology (Kirchberger *et al*. 2014). However, ML analysis of ingroup data excluding the outgroup resulted in the same overall tree topology, with taxa clustering by population within species, suggesting that the outgroup does not affect ingroup relationships (data not shown).

In contrast to the close relationships of *Alcolapia* species, we found considerably more differentiation within *O. amphimelas* between the geographically separated Lake Eyasi and Manyara populations than within *Alcolapia* (Fig. 2A), highlighting the possibility of cryptic diversity within the other soda lakes of East Africa.

### Population structure

The clusters identified in STRUCTURE analyses at optima *K* = 3 and *K* = 4 did not definitively separate intraspecific populations within Lake Natron, but do show variable levels of cluster membership by site for *A. alcalica*, with populations on the periphery of the southern Lake Natron lagoon (sites 05, 11, 12,) showing higher levels of cluster membership with sympatric species (Fig. 3). This suggests that the presence of other species prevents clean clustering by site. Meanwhile, those *A. alcalica* populations in isolated lagoons (sites 06, 09, 15, 19) exhibited minimal admixture with other species (Fig. 3). Differentiation as measured by *F*_ST_ suggested an effect of geography (Table S3, Supporting information), with pairwise population *F*_ST_ values revealing higher levels of differentiation between more distant populations, and *A. grahami* populations exhibiting the highest *F*_ST_ values when compared to Lake Natron populations. Despite these differences between sampling sites, there was no correlation between genomic and geographic distances based on Mantel tests (Table S9, Supporting information).

The *F*_ST_ values are slightly higher than, but generally show similar patterns to, those found in a recent Lake Natron study using microsatellites (Zaccara *et al*. 2014) and have similar values to those observed between differentiated cichlid populations in other recent crater lake radiations (Barluenga & Meyer 2004; Elmer *et al*. 2010b). High migration rates between lagoon populations and lack of genetic differentiation have previously been explained by the possibility of heavy rains and flooding increasing permeability of the genetic barrier created by trona crusts (e.g. Zaccara *et al*. 2014). Although even heavy floods may be insufficient to allow panmixia within the lakes, as observations report heavy *Alcolapia* mortality in floodwater between lagoons due to deoxygenation and salinity increase from dissolution of the soda deposits (Coe 1969; Tichy & Seegers 1999; Wilson *et al*. 2004). Previous studies also found morphological and physiological/behavioural differentiation between separate lagoons in Lake Magadi, suggesting local adaptation among populations (Seegers & Tichy 1999; Wilson *et al*. 2004).

Other recent cichlid radiations in which morphs exhibit differences in trophic morphology or colour have shown similarly low levels of genomic differentiation with high levels of phenotypic diversity (e.g. Barluenga & Meyer 2010). One case of divergence between Nicaraguan cichlid Lake Apoyeque morphs, and thin‐ and thick‐lipped forms of *Amphilophus cf. citrinellus* is thought to have arisen in only ~100 years (Elmer *et al*. 2010b). In the case of the Midas crater lake cichlids in particular, low levels of genomewide differentiation between ecologically divergent species and morphs have been shown to be underpinned by selection acting on a few small genomic regions (Elmer *et al*. 2010a). Outside of the cichlid radiations, a comparably young radiation with similar levels of trophic phenotypic diversity, the <10 000‐year‐old radiation of *Cyprinodon* pupfish on San Salvador Island, exhibited interspecific *F*_ST_ values of 0.12–0.49 based on RAD data (Martin & Feinstein 2014), whereas our analysis identified interspecific *F*_ST_ of only 0.04–0.20 (Table S3, Supporting information). However, the interspecific *F*_ST_ values seen within the current study are within the range of differentiation observed in adaptive divergence in other fish radiations, such as three‐spine stickleback populations (0.01–0.13, Hohenlohe *et al*. 2010; 0;.03–0.38, Jones *et al*. 2012), Lake Constance *Coregonus* species (0.02–0.08, Vonlanthen *et al*. 2012), whitefish ecotypes (0.001–0.05, Gowell *et al*. 2012), sailfin silversides (0.00–0.21, Schwarzer *et al*. 2008), and fresh and saltwater killifish (0.04–0.40, Kozak *et al*. 2013). Even lower interspecific *F*_ST_ values have been recorded between fish species differentiated mainly by colour, such as marine hamlets (*F*_ST_ = 0.0038, Puebla *et al*. 2014).

### Genomic islands of differentiation in the *Alcolapia* species flock

Despite the low overall genomic differentiation between *Alcolapia* species (Figs 2, S11, Table S3, Supporting information), our analysis supports peaks of differentiation across the genome between species (Fig. 4, Table 2). Sliding‐window analyses identified several high‐ *F*_ST_ windows distributed heterogeneously across the genome in all *Alcolapia* comparisons. This pattern was also observed in the identification of several *F*_ST_ outliers in the bayescan analysis, all of which indicated diversifying selection rather than balancing selection (Tables 2, S4–S7, Supporting information). This is consistent with a scenario of ongoing gene flow between species resulting in homogenization across the genome except for regions under divergent selection (Wu 2001; Gavrilets & Vose 2005). It is possible that these narrow regions comprise genomic islands of speciation (e.g. Turner *et al*. 2005; Nosil *et al*. 2009a; Nadeau *et al*. 2012); however, further investigation would be required to ascertain their contribution to the speciation process. Surprisingly, we find equivalent levels of divergence between the allopatric comparison of *A. alcalica*/*A. grahami* as with sympatric comparisons of *A. alcalica*/*A. latilabris* and *A. ndalalani*.

Feeding specialization leading to reproductive isolation has previously been shown to be central to speciation in fish radiations (Nosil 2012; reviewed in Bernardi 2013; Seehausen & Wagner 2014), so divergent selection acting on trophic morphology loci could lead to heterogeneous genomic differentiation. Such localized divergence has previously been observed in recently diverged sympatric cichlid species (Franchini *et al*. 2014). Although it is thought that all *Alcolapia* species currently feed on the same resources of algae and cyanobacteria (Coe 1969; Trewavas 1983), it is likely that there was more extensive trophic niche space available in the deeper palaeolake and *Alcolapia* was not restricted to shallow volcanic springs and lagoon edges. Lake depth (but not lake area) along with energy input (radiation) has been shown to be linked with propensity to diversify in cichlids (Wagner *et al*. 2012). The influence of these factors on diversification has been suggested to be a result of high carrying capacities but also short generation times and increased mutation rates, which are both thought to be factors at play within this system (Wilson *et al*. 2004). Although lake area is not associated with potential to diversify (Wagner *et al*. 2012), for those lakes in which diversification does occur, area predicts number of resultant lineages as adaptive radiation appears to scale with area (Wagner *et al*. 2014). Thus, the restricted lake area in this system may explain why the soda lake radiation contains lower species diversity than that seen in radiations from larger lakes.

### Alcolapia diversification and soda lake colonization

While the current shallow habitat depth for *Alcolapia* (maximum 0.2–1.2 m) negates the benthic–pelagic axis along which freshwater diversification is often seen (e.g. Schliewen *et al*. 2001; Vonlanthen *et al*. 2009; Wagner *et al*. 2012; Praebel *et al*. 2013; Franchini *et al*. 2014), the maximum depth of the palaeolake Orolonga (50–60 m; Roberts *et al*. 1993) was greater than the depth range over which diversification has been recorded in other shallow‐water cichlid systems (e.g. Schliewen *et al*. 2001; Seehausen *et al*. 2008). Thus, adaptation and diversification could have occurred in a deeper, oligosaline lake. In line with this reasoning, our phylogeny is consistent with a scenario in which colonization of the palaeolake occurred by a freshwater ancestor, with subsequent adaptation to saline/alkaline conditions. Within the deeper water of the palaeolake, divergence would have been possible between terminal mouth morphology (*A. alcalica*) and inferior mouth morphology (*A. ndalalani*/*A. latilabris*) along the major ecological axis of pelagic or surface feeding vs. benthic feeding (Seehausen & Wagner 2014). As the water levels dropped and lakes Natron/Magadi formed, *A. grahami* would have been geographically isolated from the remaining *Alcolapia* species, while partitioning of ecological niche and divergence of the inferior mouth morphology (thick vs. thin lips) could explain *A. latilabris* and *A. ndalalani* divergence. However, further empirical work would be required to test the ecological and functional relevance of these different trophic morphologies.

As well as being considerably smaller than Lake Natron (covering only ~20% of the area), Lake Magadi also differs in having no perennial inflowing streams, while Lake Natron has two inflowing rivers, Peninj and Ewaso Ngiro, as well as several perennial streams (Olaka *et al*. 2010). This factor not only has implications for hydrochemical variability between the two lakes, but also in terms of niche space available, as inflowing rivers and streams provide longer stretches of continuous open water than the volcanic springs. Furthermore, a previous study recorded differential species distributions along the same stream, with *A. latilabris* found more abundantly in the upper courses (Seegers *et al*. 2001), which could indicate partitioning of habitat use. As such, there may be ecological differences driving genetic differentiation between the Magadi/Natron species in addition to the allopatric separation.

### Hybridization within the *Alcolapia* radiation

Species radiations are frequently characterized by interspecific hybridization after the onset of speciation (Grant & Grant 2008). Here, we quantitatively demonstrate using *f*_*4*_ tests strong evidence for recent gene flow among all three Lake Natron *Alcolapia* species. The ML phylogeny also revealed that individuals from a single collecting locality (site 17) did not cluster by species (Fig. 2C). The two samples that exhibited an intermediate form between *A. alcalica* and *A. ndalalani* (causing difficulties with original species designation) grouped with one of the putative parental species (*A. ndalalani*). However, we may have expected the individuals identified as possible hybrids by phenotype to group separately from parental species. It is possible that, if narrow regions of the genome control traits of coloration and mouth morphology (on which species descriptions are based), hybrid individuals could possess a species‐typical phenotype of one parental species while exhibiting a combination of both parental genotypes across the rest of the genome. However, we do not test this hypothesis in our current analysis. Furthermore, while previous studies have shown hybridization to be an important mechanism in the diversification of other cichlid lineages (Seehausen 2004), additional work would be required to test its role within the soda lake system.

If the described species diverged in the larger palaeolake environment, then it remains to be seen whether these species are sufficiently reproductively isolated to maintain separation in the contracted niche space of the shallow springs. Experimental testing of reproductive isolation in this system would provide information on the completeness of speciation (e.g. Nosil *et al*. 2009b) Furthermore, incipient species may never achieve full speciation if the speciation process is reversed by interspecific hybridization brought on by changes in the environment. However, it seems unlikely that *Alcolapia* are currently undergoing speciation reversal given that we found similar frequencies of outlier SNPs in the sympatric Lake Natron species comparisons as in the allopatric *A. alcalica*/*A. grahami* comparison (Table 2). If speciation reversal were occurring, we would expect the sympatric species to show fewer putative outlier SNPs under diversifying selection than the allopatric species, as introgression would erode peaks of differentiation. Such an impact of hybridization has been seen in European whitefish where a breakdown in reproductive isolating mechanisms increased gene flow between species, reducing the extent of genomic islands of differentiation, and exhibiting fewer candidate outlier loci (Vonlanthen *et al*. 2012; Hudson *et al*. 2013).

### *Alcolapia* species flock as a study system for speciation

The *Alcolapia* flock represents a young, small‐scale radiation with several incipient species. Although the present study demonstrates that the *Alcolapia* lineages may not be strongly reproductively isolated, with significant levels of admixture between species (Table 1), they may be considered species under the genotypic clusters species concept (Mallet 1995). Given the recent diversification of the species flock, there is likely to be some level of incomplete lineage sorting, and yet nearly all samples in our analysis sort by species first, and then sampling site, suggesting there are existing segregating sites between species. It seems unlikely that these differences merely describe population differentiation (via drift or local adaptation) given that divergence is maintained in geographic contact (deemed a ‘critical test’ of incipient speciation, Seehausen & Wagner 2014). Furthermore, the occurrence of ongoing gene flow and admixture in founding populations is increasingly being found in adaptive radiations (e.g. Lamichhaney *et al*. 2015), and several fish radiations are thought to have emerged from a ‘hybrid swarm’ origin (Seehausen 2004; Hudson *et al*. 2011). As such, the *Alcolapia* species flock appears to be at a very early stage of speciation and offers an excellent system to investigate processes generating biodiversity. Incipient species may be the most useful for examining generation of reproductive isolation, where barriers that contributed to speciation (rather than arose after speciation was complete) can be tested (Coyne & Orr 2004).

Finally, colonization inference may be more straightforward in this young and geographically restricted system than in larger water bodies such as the African Great Lakes with older radiations and greater species diversity. The endemism of *Alcolapia* within the Natron/Magadi basin means that repeated colonization scenarios or continuing introgression from external sources (e.g. Schliewen *et al*. 2006) is unlikely. However, the fragmentary nature of the soda lake environment and fluctuations in water levels could periodically impact population connectivity, complicating the inference of sympatry over past timescales.

The unique physiological adaptations to extreme environmental conditions in *Alcolapia*, their endemism and geographic exclusion from other *Oreochromis* species, as well as a fragile ecosystem sensitive to anthropogenic change, create a conservation priority for these fishes. All *Alcolapia* species are categorized as endangered or vulnerable on the IUCN red list (Bayona 2006; Bayona & Akinyi 2006), and populations are potentially threatened by planned development of an ash mining plant at Lake Natron with concurrent development of water extraction and infrastructure (Kadigi *et al*. 2012).

Our findings, which demonstrate recent divergence, ongoing gene flow, and low levels of genomewide divergence alongside narrow peaks of high divergence certainly warrant further investigation to elucidate the processes initiating and maintaining speciation in this system. A clear future research goal would be to identify regions of the genome that are responsible for the phenotypic diversification and polymorphism observed in these cichlid fishes, despite the very shallow divergence between species. Although the present study highlights heterogeneous genomic differentiation, more detailed analysis is required to identify the regions of high divergence and assess their impact on species differentiation.

## Funding

This work was supported by a BBSRC/NERC SynTax grant (JJD and KG), UCL Graduate Scholarship and Graduate School Research Project Fund Award (AGPF), and Genetics Society training grant (AGPF), and the Percy Sladen Memorial Fund (JJD).

All authors designed the study. A.G.P.F., J.J.D. and L.R. conducted fieldwork and collected samples. A.G.P.F., K.K.D., T.C. and K.G. conducted laboratory work and data analysis. A.G.P.F., J.J.D., L.R. and K.K.D. wrote the manuscript. All authors reviewed and contributed to editing of the manuscript.

## Data accessibility

The demultiplexed, unaligned Illumina sequence data in fastq format have been uploaded to ENA (Accession no.: ERP009428).

The raw vcf files for reference‐aligned and de novo‐assembled reads, the quality‐filtered genotype alignment files and all tree files presented in this manuscript have been deposited at http://datadryad.org/ under the doi:10.5061/dryad.s01kf.

Sampling locations and population information are given in the Supplementary Information (Table S1, Supporting information).

## Supporting information


**Table S1.** Collection coordinates and sequencing statistics per sample.
**Table S2**. Data subsets and respective analyses conducted on RAD data.
**Table S3.** Population pairwise *F*_ST_.
**Table S4.** bayescan outlier loci for *A. alcalica* vs. *A. grahami* comparison.
**Table S5.** bayescan outlier loci for *A. alcalica* vs. A. *latilabris* comparison.
**Table S6.** bayescan outlier loci for *A. alcalica* vs. *A. ndalalani* comparison.
**Table S7.** bayescan outlier loci for *A. latilabris* vs. *A. ndalalani* comparison.
**Table S8.** Gene annotations of loci identified as outliers in multiple species comparisons.
**Table S9.** Mantel test results for *F*_ST_ vs. geographic distance between sampling sites.

**Fig. S1.** Catchment area of the Natron–Magadi basin.
**Fig. S2.** Plots of linkage disequilibrium dropoff with distance by species.
**Fig. S3.** ML phylogeny for variable sites using the ASC_model.
**Fig. S4.** Majority consensus (50%) ML phylogenies generated from additional RAD data sets.
**Fig. S5.** Species tree generated by SNAPP analysis for selected populations.
**Fig. S6.** Visualization of *K* = 2–5 for *Alcolapia* STRUCTURE analysis.
**Fig. S7.** Visualization of *K* = 2–5 for sympatric *Alcolapia* STRUCTURE analysis.
**Fig. S8.** Visualization of *K* = 2–5 for *A. alcalica* STRUCTURE analysis.
**Fig. S9.** Frequency histograms of sliding‐window *F*_ST_ values for pairwise comparisons.
**Fig. S10.** Highest scoring windows in the nonoverlapping window *F*_ST_ analysis.
**Fig. S11.** Mean interspecimen uncorrected p‐distance for filtered RAD data from aligned and de novo‐assembled reads.
