# Supplementary material for: High levels of interspecific gene flow in an endemic cichlid fish adaptive radiation from an extreme lake environment
Source: Mol Ecol. 2015 Jun 19;24(13):3421–40. doi: 10.1111/mec.13247 (PMC4973668; doi:10.1111/mec.13247)
Supplement: Supplementary file 2 — Fig. S1. Catchment area of the Natron–Magadi basin. Fig. S2. Plots of linkage disequilibrium dropoff with distance by species. Fig. S3. ML phylogeny for variable sites using the ASC_model. Fig. S4. Majority consensus (50%) ML phylogenies generated from additional RAD data sets. Fig. S5. Species tree generated by SNAPP analysis for selected populations. Fig. S6. Visualization of K = 2–5 for Alcolapia STRUCTURE analysis. Fig. S7. Visualization of K = 2–5 for sympatric Alcolapia STRUCTURE analysis. Fig. S8. Visualization of K = 2–5 for A. alcalica STRUCTURE analysis. Fig. S9. Frequency histograms of sliding‐window F ST values for pairwise comparisons. Fig. S10. Highest scoring windows in the nonoverlapping window F ST analysis. Fig. S11. Mean interspecimen uncorrected p‐distance for filtered RAD data from aligned and de novo‐assembled reads. [file MEC-24-3421-s002.pdf]

Extensive gene flow among soda lake cichlid species

Supplementary Information

Supplementary Information table of contents **1**
SI Methods **2**
RAD library construction 2
Estimation of the extent of linkage disequilibrium 2
Additional STRUCTURE analysis 3
SI Results **3**
Generation of a genome-wide SNP dataset using RAD Sequencing 3
Additional STRUCTURE analysis 4
Effect of data quality filtering on phylogenomic signal 4
Phylogenomic inference using next generation sequencing (NGS) datasets 4
SI References **6**
SI Figures **7**
Figure S1. Catchment area of the Natron-Magadi basin. 7
Figure S2. Plots of linkage disequilibrium dropoff with distance by species. 8
Figure S3. ML phylogeny for variable sites using ASC_model. 9
Figure S4. ML phylogenies generated from additional RAD datasets. 10
Figure S5. Species tree by SNAPP analysis for selected populations. 11
Figure S6. Visualisation of K=2-5 for *Alcolapia* STRUCTURE analysis. 12
Figure S7. Visualisation of K=2-5, sympatric *Alcolapia* STRUCTURE analysis 13
Figure S8. Visualisation of K=2-5 for *A. alcalica* STRUCTURE analysis. 14
Figure S9. Frequency histograms of sliding-window F_{ST} values 15
Figure S10. Highest scoring windows in the sliding-window F_{ST} analysis 16
Figure S11. Mean inter-specimen uncorrected p-distance. 17
SI Tables **18**
Table S1. Collection co-ordinates and sequencing statistics per sample. 18
Table S2. Data subsets and respective analyses conducted on RAD data 21
Table S3. Population pairwise F_{ST}. 22
Table S4. Bayescan outlier loci for *A. alcalica* vs *A. grahami* comparison. 23
Table S5. Bayescan outlier loci for *A. alcalica* vs *A. latilabris* comparison........ 24
Table S6. Bayescan outlier loci for *A. alcalica* vs *A. ndalalani* comparison. 25
Table S7. Bayescan outlier loci for *A. latilabris* vs *A. ndalalani* comparison. 26
Table S8. Outlier SNPs and predicted gene annotations. 28
Table S9. Mantel test results. 29

SI Methods

RAD library construction

Phenol/Chloroform extraction protocol, modified from (Sambrook & Russel 2001)

1. Place tissue sample in 1.5mL Eppendorff tube with 500µl extraction buffer with SDS (recipe below) and 15µL Proteinase K
2. Incubate at 37˚C for 6 hours or 55˚C for 4 hours. [RAD: 55˚C for 1 hour]
3. Vortex and spin down
4. Add 4µL RNAse A and incubate for 2 minutes
5. Mix by overhead inversion for 10 minutes
6. Add 75µL NaCl 5M and mix by inversion for 10 mins
7. Add 650µL Phenol:Chloroform:Isoamyl Alcohol (25:24:1) – in fume cupboard
8. Mix by overhead inversion for 10 mins
9. Centrifuge: 15 mins at 10,000 RPM
10. Remove supernatant phase to new tube – in fume cupboard
11. Add 1000µL ice cold 100% ethanol (stored in freezer)
12. Put samples in freezer -20 ˚C for >1 hour
13. Centrifuge: 15 mins at 15,000 RPM at 4 ˚C
14. Pour off ethanol (being careful not to dislodge pellet at bottom of tube)
15. Add 1500µL ice cold 70% ethanol (from freezer)
16. Centrifuge: 10 mins at 13,000 RPM 4 ˚C
17. Pour off ethanol (taking care not to dislodge pellet)
18. Dry samples on hot blocks at 37 ˚C for ~15 mins
19. Resuspend pellet in 50-75µL distilled H20 or DNA storage buffer (TE)

Extraction buffer (100 mL)

- 10mL Tris-HCl 1M (pH=8)
- 2mL EDTA 0.5M
- 2mL NaCl 5M
- 0.5mL SDS 20%
- 85.5mL dH_20

Estimation of the extent of linkage disequilibrium

We estimated LD for each species using the R package snpstats (Clayton & Leung 2007), (with R scripts modified from (Martin *et al.* 2013)) using the reference-aligned dataset. We estimated LD for pairs of SNPs within each linkage group, and then averaged the correlation coefficient r^2 across all linkage groups within pre-specified distance bins. We did not include in our calculations any scaffolds that were not assigned to specific linkage groups of the reference genome (i.e., those positions within the genome designated as unknown scaffolds, which accounted for 22% of our full alignment; dataset C, table S2). Varying the values of the MAF threshold and the allowed limit of missing data, as well as the number of individuals included per species, had large effects on the dropoff rate of LD, so we used conservative thresholds in our final analysis. LD was estimated for each species separately using only SNPs from the aligned dataset with a minimum allele frequency (MAF) of 0.2 and for sites at which there was data for a minimum of 80% of individuals. We present LD dropoff plots for analysis using 12 individuals per species for all the *Alcolapia* species. Additionally, for *A. grahami* we include a plot for n=8 which includes only the Lake Magadi samples, and excludes those from the introduced population in Lake Nakuru. Given the apparent divergence between lakes for *O. amphimelas*, we present only the analysis of LD dropoff

for *O. amphimelas* samples from Lake Eyasi (n=4); although including the additional samples from Lake Manyara had little impact on the result (data not shown).

Additional STRUCTURE analysis

In addition to the STRUCTURE analysis of the entire Alcolapia dataset described in the main manuscript, we also conducted analyses on subsets of the data. As the full-dataset STRUCTURE runs indicated differences in cluster membership between allopatric and sympatric sites, we also conducted STRUCTURE analysis on a dataset containing only individuals occurring sympatrically in Lake Natron (sites 005 and 011) and ran these analyses separately and combined, with a minimum of 500kb between SNPs and the following parameters: site 05: 12 individuals; 2,180 SNPs; λ=0.8; K=1-8; site 11: 12 individuals; 2,160 SNPs; λ=0.8; K=1-8; site 05 and 11 combined: 24 individuals; 2,227 SNPs; λ=1.1; K=1-16.

As the *Alcolapia* dataset indicated separation within the A. alcalica samples (see Results section), we also repeated the analysis on a separate dataset containing only A. alcalica samples (38 samples, 2,222 SNPs), with an obtained value of λ=0.7184, for five independent runs at each K value of K=1–8.

SI Results

Generation of a genome-wide SNP dataset using RAD Sequencing

A total of 83.6 Gb of sequence was produced, of which 89% successfully mapped to the *O. niloticus* reference genome in the alignment stage. Mapping rates were generally consistent across species, although slightly lower for *A. grahami* than the other species (mean mapping rates: *O. amphimelas*: 89.9%; *A. alcalica*: 88.8%; *A. latilabris*: 88.7%; *A. ndalalani*: 88.8%, *A. grahami*: 86.7%). Individual mapping rates per sample are given in Table S1. Following removal of duplicates and poorly paired reads, the dataset comprised 272,360,470 paired reads. From these filtered reads, a total of 1,229,734,617 calls were generated using the GATK UnifiedGenotyper (mean: 12,809,736; range 313,244–21,316,544 calls). Five samples with fewer than 2.5 million calls were removed from further analysis, resulting in a dataset of 91 samples (84 *Alcolapia* species and 7 *O. amphimelas*). Quality filtering (see *Methods* for specific parameters) resulted in a final dataset of 28,560,699 retained sites, representing ~3% of the reference genome. The final dataset comprised 544,916 sites that were variable across the whole alignment including *O. niloticus* (1.91%), of which 238,203 sites were variable across *Alcolapia* samples and 164,014 sites were variable across *A. alcalica* samples alone.

A separate dataset was compiled from reads that did not map to the *O. niloticus* reference genome, as such reads are likely to lie in more divergent regions of the genome. After quality filtering as for the mapped reads, the dataset contained a total of 436,839 sites, of which 5,832 (1.34%) were variable across the 91-sample dataset. The combined dataset of mapped and unmapped reads resulted in a total alignment of 550,748 variable sites (fewer than that from the mapped dataset alone as the *O. niloticus* reference sites were removed before combining).

Additional STRUCTURE analysis

The STRUCUTRE analysis for the full dataset (presented in the main paper) gave optima for the K value of K=3 and K=4. When the Lake Natron sympatric sites were analysed separately (Figure S7), both site 05 and site 011 had an lnP(K) optimum of K=1 and an Evanno method optimum at K=2, but the cluster membership differed between sites, with the site 05 analysis indicating most likely membership to a single cluster across all species, while site 011 indicated different cluster membership proportions by species. When analysed jointly, optima were seen at K=2 and K=4, with the latter K value indicating differing cluster membership between species, and between populations with *A. alcalica* (Figure S7). Given the separation of northern and southern *A. alcalica* populations indicated by both the phylogenetic inference and STRUCTURE analyses, we also conducted STRUCTURE analysis on a dataset including only the *A. alcalica* samples (dataset Q) in order to disentangle population clustering within this species. Replicate runs gave an optimum of K=1, suggesting that populations within *A. alcalica* are not sufficiently diverged to detect population structure within the species at this scale (Figure S8).

Effect of data quality filtering on phylogenomic signal

As in previous analyses of very young cichlid radiations (Wagner *et al.* 2013), we found that inclusion of maximal data from RAD sequencing gave the best phylogenomic resolution, and that removal of additional data in filtering and QC steps (e.g., imposing thresholds for maximum amount of missing data, using higher SNP quality thresholds or higher threshold for individual inclusion) resulted in poorer phylogenomic resolution in ML analysis. This reflects phylogenetic analysis of simulated RAD data, showing that missing data do not appear to negatively impact phylogenetic accuracy, but low levels of informative data may do so (Rubin *et al.* 2012). Thus, we finally used a dataset imposing minimal threshold for missing data (including all sites with data for >2 individuals), a moderate SNP quality threshold (20) and removed only those individuals with considerably lower sequencing quality than the rest of the dataset (<2.5 million sequenced reads; a total of five individuals). While it may seem intuitively preferable to use maximal stringency in filtering to ensure optimally clean data, several recent analyses have revealed that such stringency may obscure phylogenomic signal in noisy datasets, removing important information in the form of outlier data (Huang & Knowles 2014), and that increased stringency in filtering thresholds may reduce accuracy of downstream analyses e.g., (Hoffman *et al.* 2014)

Phylogenomic inference using next generation sequencing (NGS) datasets

Despite the considerable increase in phylogenetically informative data from NGS, difficulties remain in finding the best way to analyse such large datasets. Of note, the RAD methodology was originally developed for population-level studies (Baird *et al.* 2008; Hohenlohe *et al.* 2010) and have only recently begun to be used in phylogenetic analysis. The more recent studies that investigate phylogeny using RAD data have typically employed phylogenomic inference using matrices of concatenated sequence data from resultant RAD

loci rather than using SNP datasets (e.g.,(Wagner *et al.* 2013). Phylogenetic algorithms have typically been developed for sequence data and do not handle concatenated SNP data well as it contains no invariant sites (Bertels *et al*. 2014), and may violate assumptions of rate heterogeneity, especially as large datasets often preclude the use of partitioning programs to assign different substitution models to different regions. Using the entire sequence data from RAD sampling (rather than only the variable sites) becomes computationally intensive with increase in taxon number, and it seems that taxa number rather than alignment length is the limiting factor in such analysis. For example, our reduced-taxon dataset (n=25; 26 million bp) took 3 days to complete analysis, whereas the full-taxon dataset (n=92; 28 million bp) would have taken >6 months to complete.

Thus, using only variable sites that are phylogenetically informative seems an intuitive way by which to reduce the dataset to feasible size. However we found that using a SNP-only dataset reduced the confidence of ML branch partitions, and at least one species-level node (*A. grahami*) was unable to be placed with any confidence, even when using the RAxML ASC_ model (ascertainment bias correction) suggested for SNP datasets, while this node was robustly resolved in the analysis of the full sequence data (Figure 2 in the main text).

As our reduced-taxon dataset was assembled from the individuals with highest sequence quality in each population, we also checked that levels of missing data in the full-taxon SNP-only dataset were not impacting results. However, imposing maximum thresholds of missing data in SNP datasets (maximum 50% and 0% missing data) did not increase bootstrap support or resolve species relationships more robustly (results not shown).

It has been suggested that ascertainment bias correction may not be appropriate for SNP alignments (Pettengill *et al.* 2014), although employing GTRGAMMA with and without the ASC model in our dataset produced very similar results (Figures 2C and S3). Though it should be noted that the results are not directly comparable as these models were run on different datasets given the removal of ambiguous bases from the alignment for use of the ASC_ model, as the ASC_ model will not run on alignments containing invariant sites, and sites that contain ambiguous bases are considered invariant if the ambiguous base could be determined to be the same as non-ambiguous bases at that site, and there are no other polymorphic bases at that site (RAxML 8 manual). While ambiguous bases in Sanger-produced sequences typically indicate lack of confidence in base-calling rather than genuine heterozygosity, those in NGS datasets are usually called only if reaching a minimum coverage in both alternative bases and so are more likely represent a heterozygous locus, and so certain assumptions from existing phylogenetic models may well be unsuited to such SNP datasets.

While we highlight some of the difficulties here in the use of NGS data for phylogenetic purposes, it is undoubtedly the case that these datasets have already provided resolution to previously unanswered questions, and the continued updating of existing phylogenetic models with SNP-only datasets, along with the development of SNP-specific protocols (e.g., SNAPP program used in our analysis) will continue to refine and enhance the inferences that can be made from such datasets.

SI References

Baird NA, Etter PD, Atwood TS *et al.* (2008) Rapid SNP discovery and genetic mapping using sequenced RAD markers. *PLoS ONE*, **3**, e3376.

Bertels F, Silander OK, Pachkov M, Rainey PB, van Nimwegen E (2014) Automated reconstruction of whole-genome phylogenies from short-sequence reads. *Molecular Biology and Evolution*, **31**, 1077–1088.

Clayton D, Leung H-T (2007) An R package for analysis of whole-genome association studies. *Human Heredity*, **64**, 45–51.

Hoffman JI, Simpson F, David P *et al.* (2014) High-throughput sequencing reveals inbreeding depression in a natural population. *Proceedings of the National Academy of Sciences of the United States of America*, **111**, 3775–3780.

Hohenlohe PA, Bassham S, Etter PD *et al.* (2010) Population genomics of parallel adaptation in threespine stickleback using sequenced RAD tags. *PLoS Genetics*, **6**, e1000862.

Huang H, Knowles LL (2014) Unforeseen consequences of excluding missing data from next-generation sequences: simulation study of RAD sequences. *Systematic Biology*, **0**, 1–9.

Martin SH, Dasmahapatra KK, Nadeau NJ *et al.* (2013) Genome-wide evidence for speciation with gene flow in *Heliconius* butterflies. *Genome Research*, **23**, 1817–1828.

Pettengill JB, Luo Y, Davis S *et al.* (2014) An evaluation of alternative methods for constructing phylogenies from whole genome sequence data: a case study with *Salmonella*. *PeerJ*, **2**, e620.

Roberts N, Taieb M, Barker P *et al.* (1993) Timing of the Younger Dryas event in East Africa from lake level changes. *Nature*, **366**, 146–148.

Rubin BER, Ree RH, Moreau CS (2012) Inferring phylogenies from RAD sequence data (S-O Kolokotronis, Ed,). *PLoS ONE*, **7**, e33394.

Sambrook J, Russel DW (2001) Commonly Used Techniques in Molecular Cloning Co. In: *Molecular Cloning*. Cold Spring Harbor Laboratory Press, Cold Spring Harbor, NY, USA.

Wagner CE, Keller I, Wittwer S *et al.* (2013) Genome-wide RAD sequence data provide unprecedented resolution of species boundaries and relationships in the Lake Victoria cichlid adaptive radiation. *Molecular Ecology*, **22**, 787–798.

SI Figures

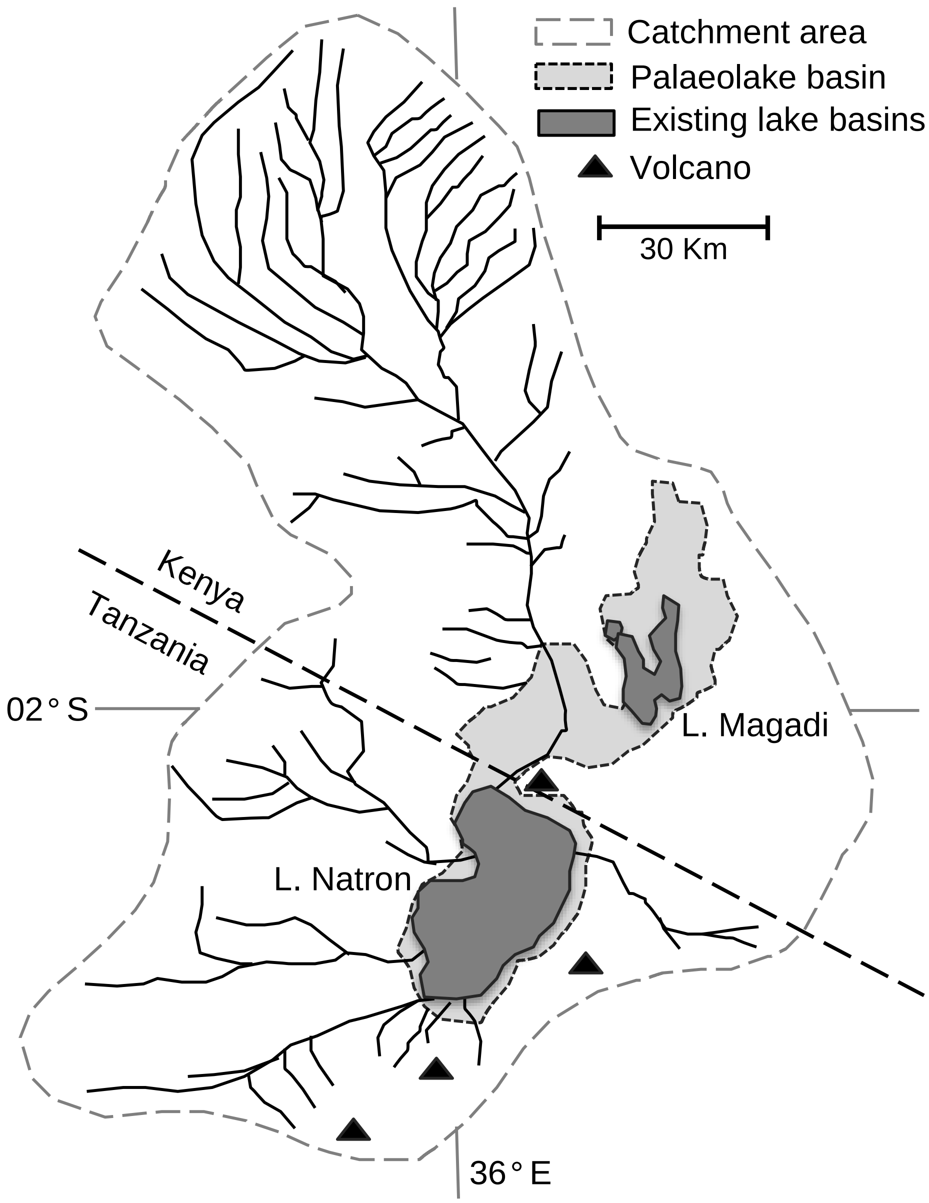

Figure S1. Catchment area of the Natron-Magadi basin.
Hydrological system and extent of palaeolake and modern lake boundaries. Modified from (Roberts *et al.* 1993).

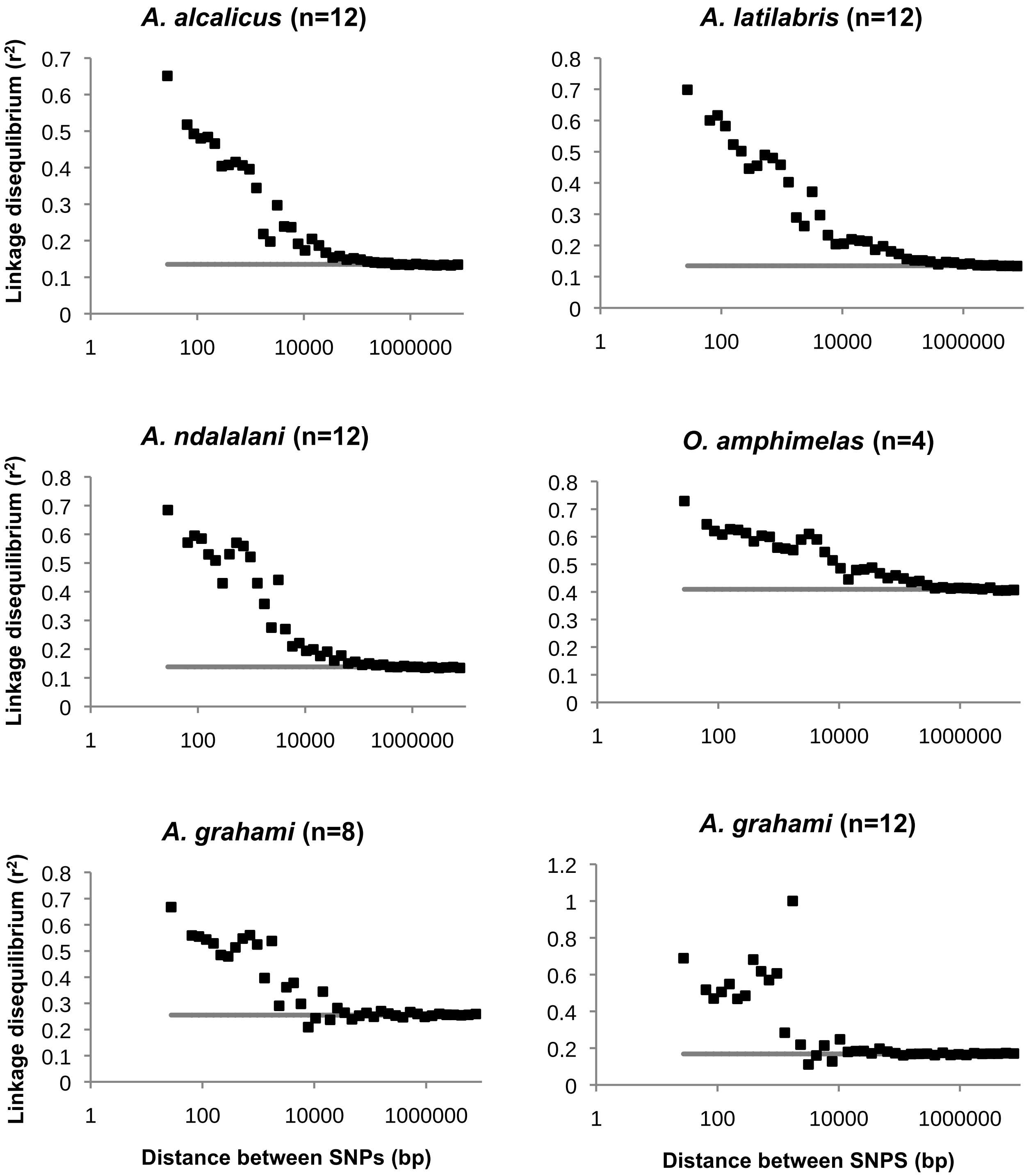

Figure S2. Linkage disequilibrium dropoff with distance by species.
Grey line in each plot indicates background LD level.

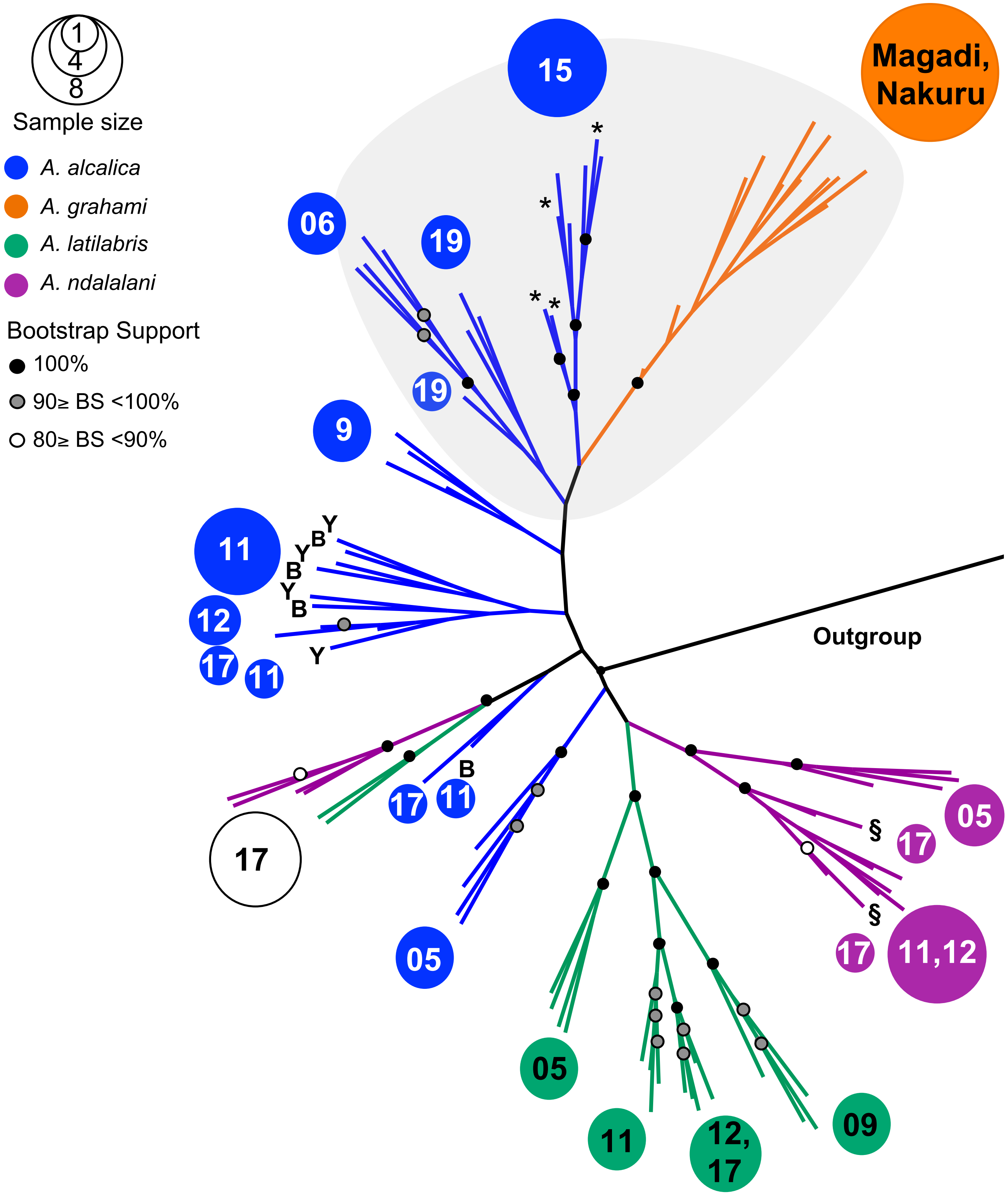

Figure S3. ML phylogeny for variable sites using ASC_model.
Full taxon dataset (n=92) alignment of variable sites only with ambiguous bases removed (dataset E; 363,983 SNPs) and ascertainment bias correction (ASC_ model) applied within RAxML. The phylogeny exhibits similar topology as the phylogeny produced when the ML analysis is conducted without the _ASC model (Figure 2C in the main paper).

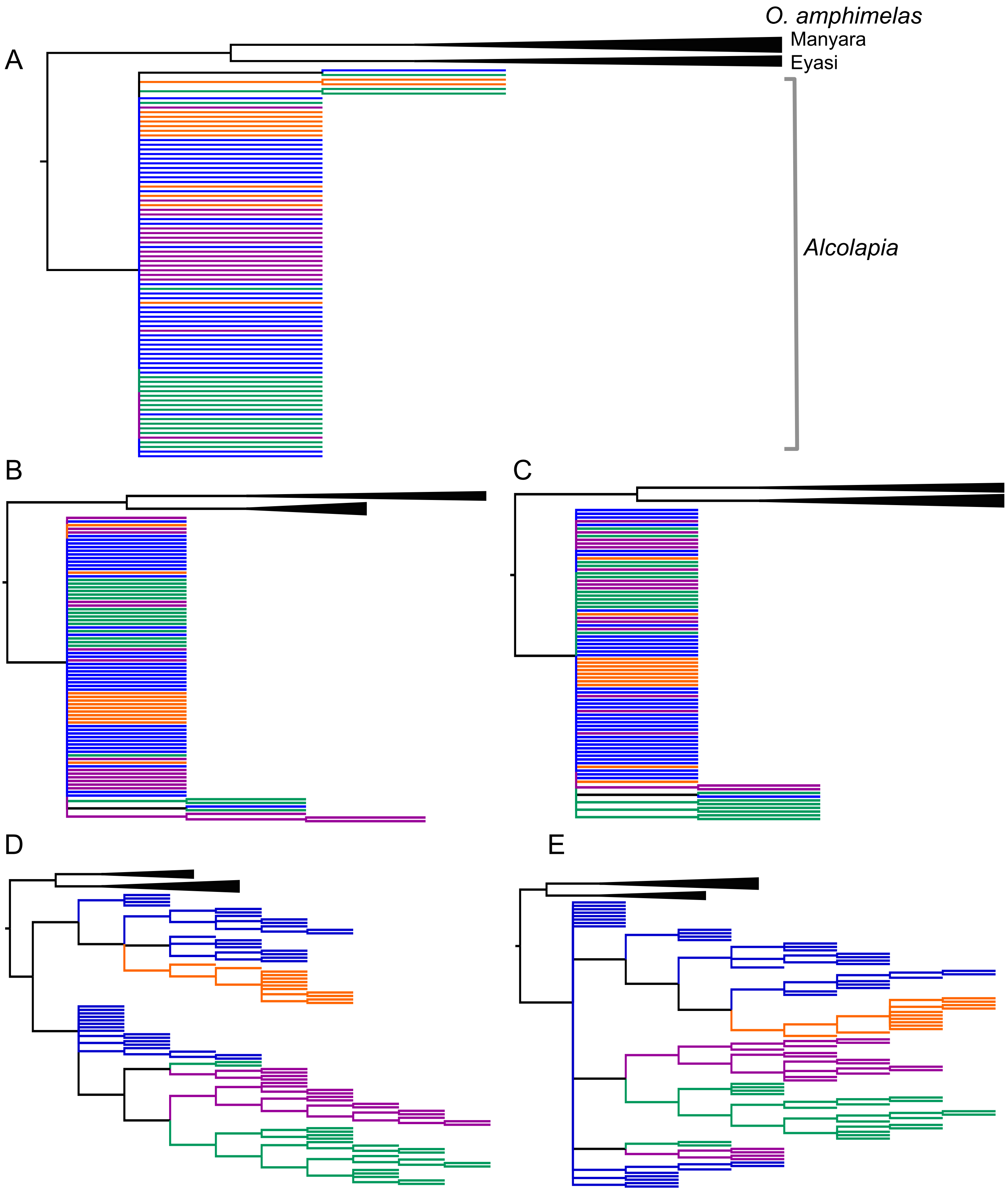

Figure S4. Majority consensus (50%) ML phylogenies generated from additional RAD datasets. For all analyses, trees are rooted using *O. amphimelas* as outgroup. A) de novo assembly (unmapped reads) full dataset (total alignment of 436,839 bp, including invariant sites); B, C) de novo assembly variable sites only (SNPs) dataset, B) all SNPs, without ASC_ correction (5,832 SNPS); C) ambiguous bases removed and employing ASC_ correction (1,898 SNPs); D, E) combined dataset of SNPs from mapped and de novo-assembled reads; D) all SNPs, without ASC_ correction (550,748 SNPS); C) ambiguous bases removed and employing ASC_ correction (182,909 SNPs).

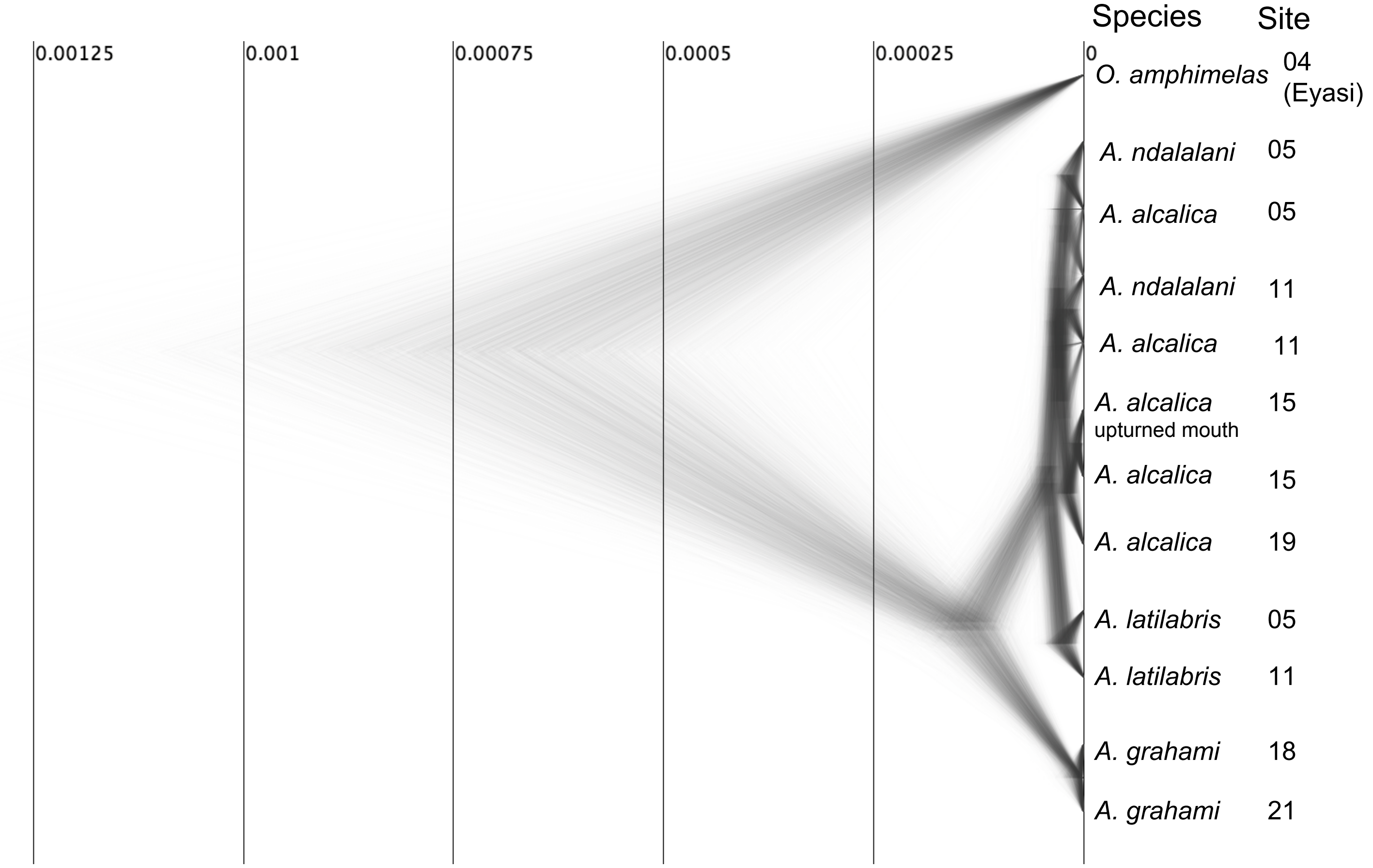

Figure S5. Species tree generated by SNAPP analysis for selected populations. Analysis for a reduced-taxon dataset of 4 individuals from each of 12 populations, with unlinked biallelic SNPs (dataset F; n=48; 1,266 SNPs).

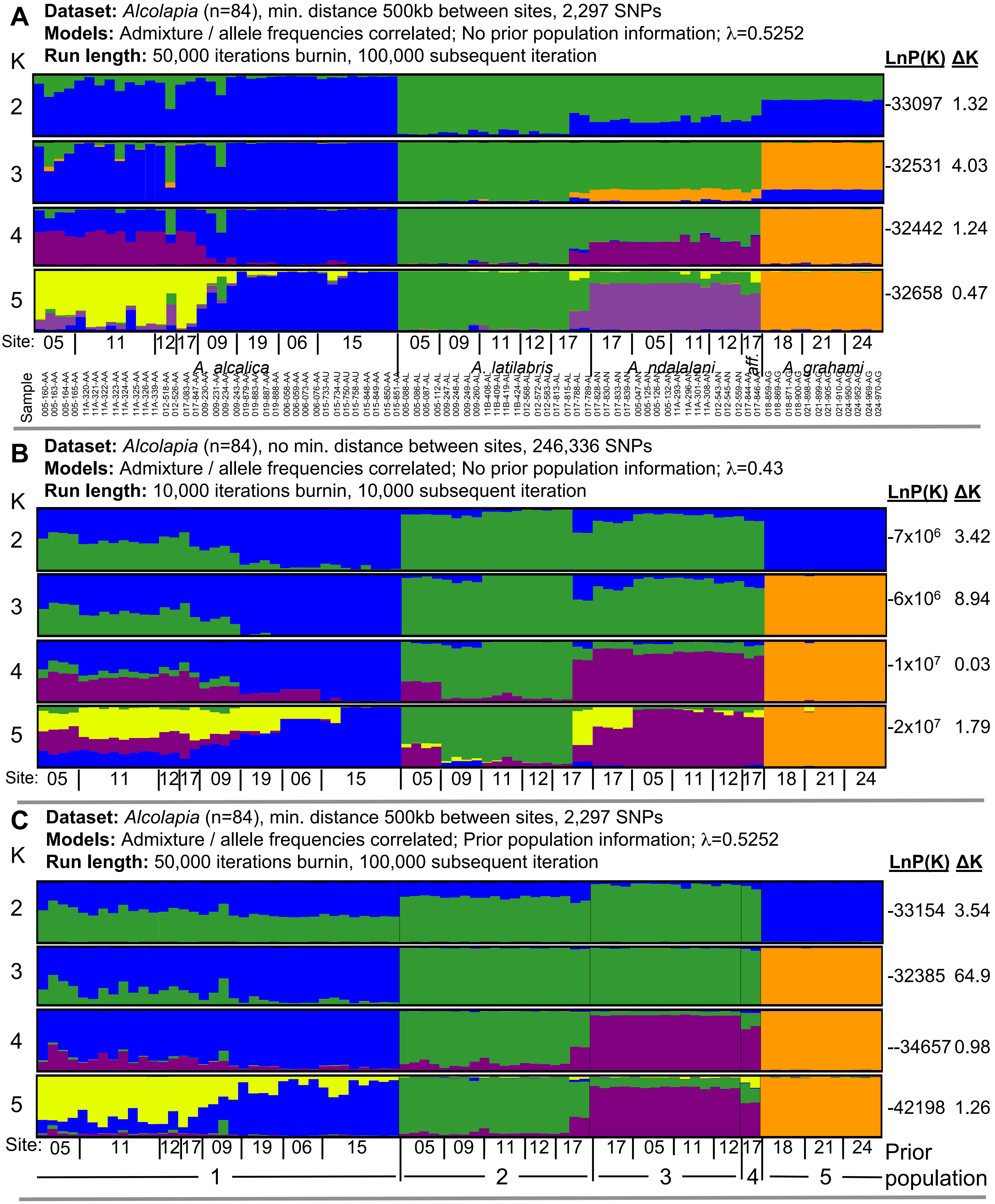

Figure S6. Visualisation of K=2-5 for *Alcolapia* STRUCTURE analysis. Cluster membership for admixture/correlated allele frequency models. A) Reduced dataset accounting for LD; B) Full dataset, no min. distance between SNPs; C) Population information included as a model prior. Samples are ordered as in panel A. LnP(K): Estimated Ln Prob of Data. ΔK: second order rate of change of the likelihood function with respect to K, the modal value of which indicates the best estimate of K.

A

Dataset: Lake Natron *Alcolapia* sites 5 and 11 only (n=24), 500kb between sites, 2,227 SNPs
Models: Admixture / allele frequencies correlated; No prior population information; λ=0.8
Run length: 50,000 iterations burnin, 100,000 subsequent iterations

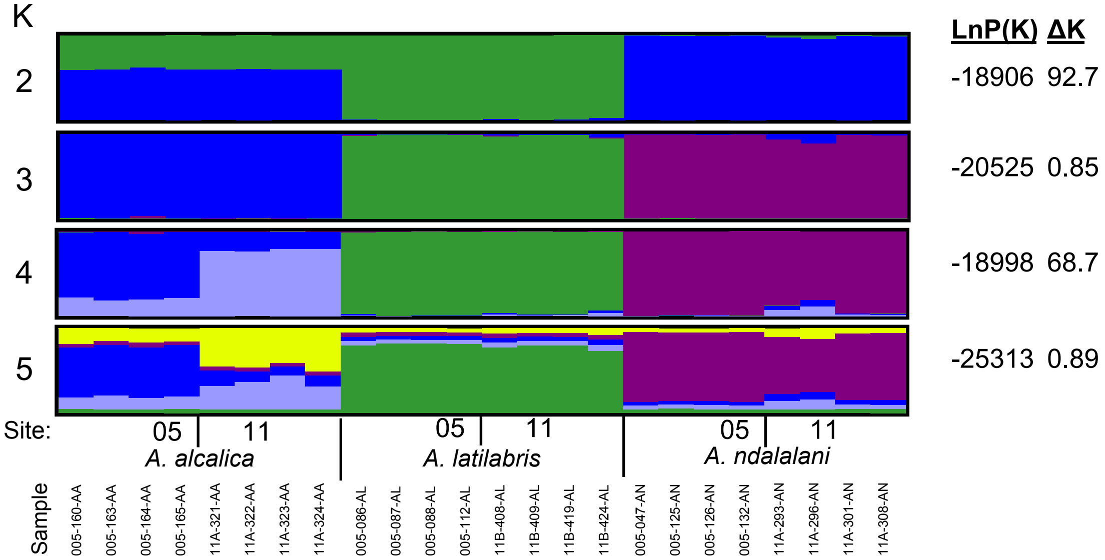

B

Dataset: Lake Natron *Alcolapia* site 5 only (n=12), 500kb between sites, 2,180 SNPs
Models: Admixture / allele frequencies correlated; No population information; λ=1.1
Run length: 50,000 iterations burnin, 100,000 subsequent iterations

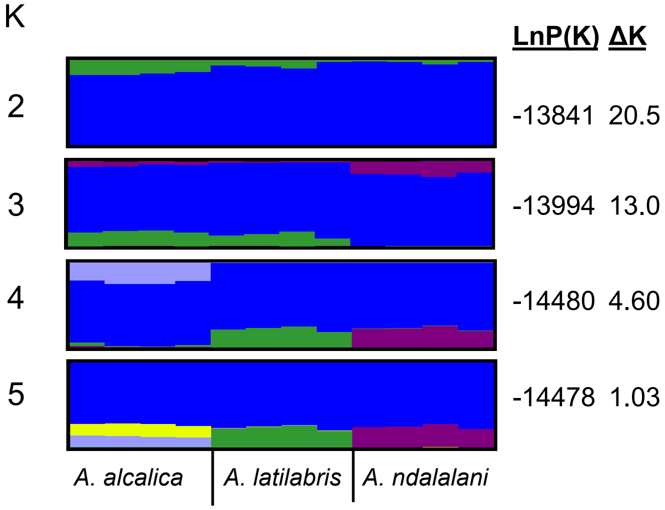

C

Dataset: Lake Natron *Alcolapia* site 11 only (n=12), 500kb between sites, 2,160 SNPs
Models: Admixture / allele frequencies correlated; No population information; λ=0.1.1
Run length: 50,000 iterations burnin, 100,000 subsequent iterations

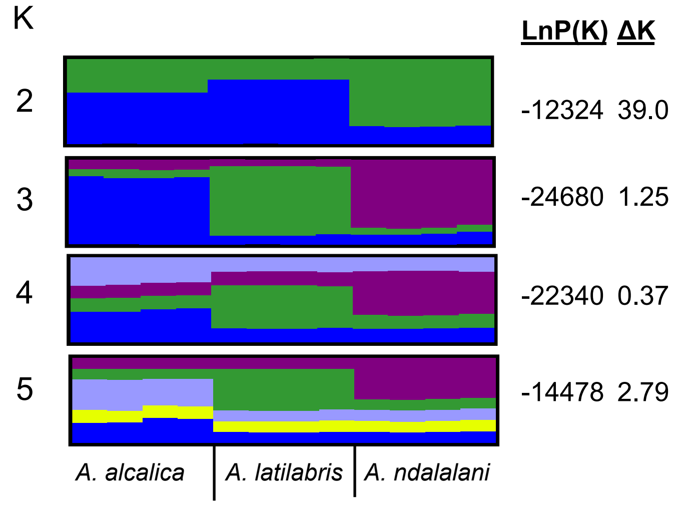

Figure S7. Visualisation of K=2-5 for *Alcolapia* STRUCTURE analysis
Cluster membership by individual for admixture / correlated allele frequency models for sites in which all three Lake Natron species occur sympatrically: A) Sites 005 and 011 Individuals combined; B) Site 005 only; C) Site 011 only.

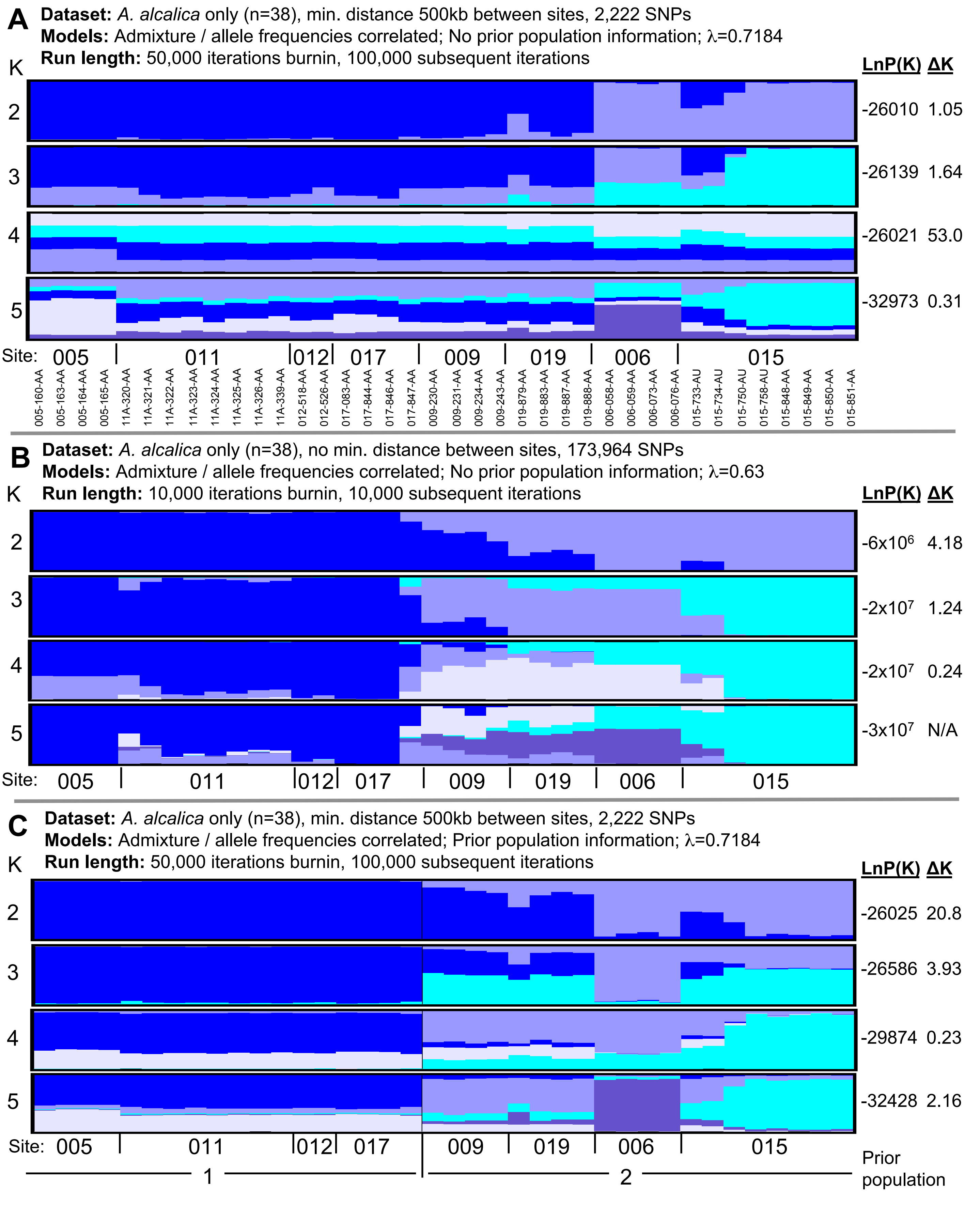

Figure S8. Visualisation of K=2-5 for *A. alcalica* STRUCTURE analysis. Cluster membership for admixture/ correlated allele frequency models. A) Reduced dataset accounting for LD; B) Full dataset with no min. distance between SNPs; C) Providing population information as a model prior. Samples ordered as indicated in panel A.

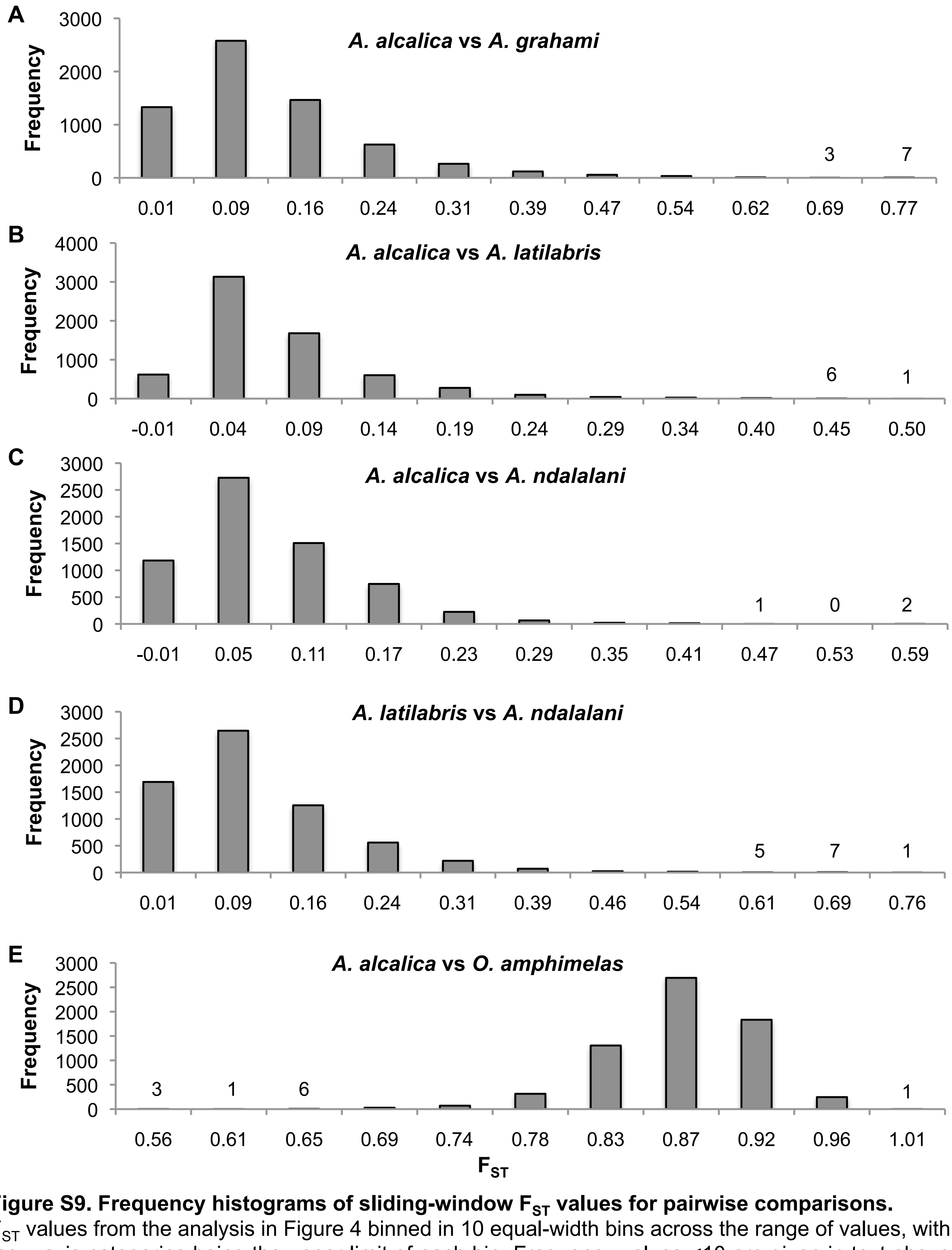

Figure S9. Frequency histograms of sliding-window F_{ST} values for pairwise comparisons. F_{ST} values from the analysis in Figure 4 binned in 10 equal-width bins across the range of values, with the x-axis categories being the upper limit of each bin. Frequency values <10 are given in text above respective columns. All within-*Alcolapia* comparisons (A-D) show a right-skewed pattern with a majority of windows showing low differentiation, but a small number showing comparatively high F_{ST} values. Conversely, the *Alcolapia*-Outgroup comparison (E) shows a left-skewed distribution, with most comparisons showing high levels of differentiation and only a few regions of low differentiation.

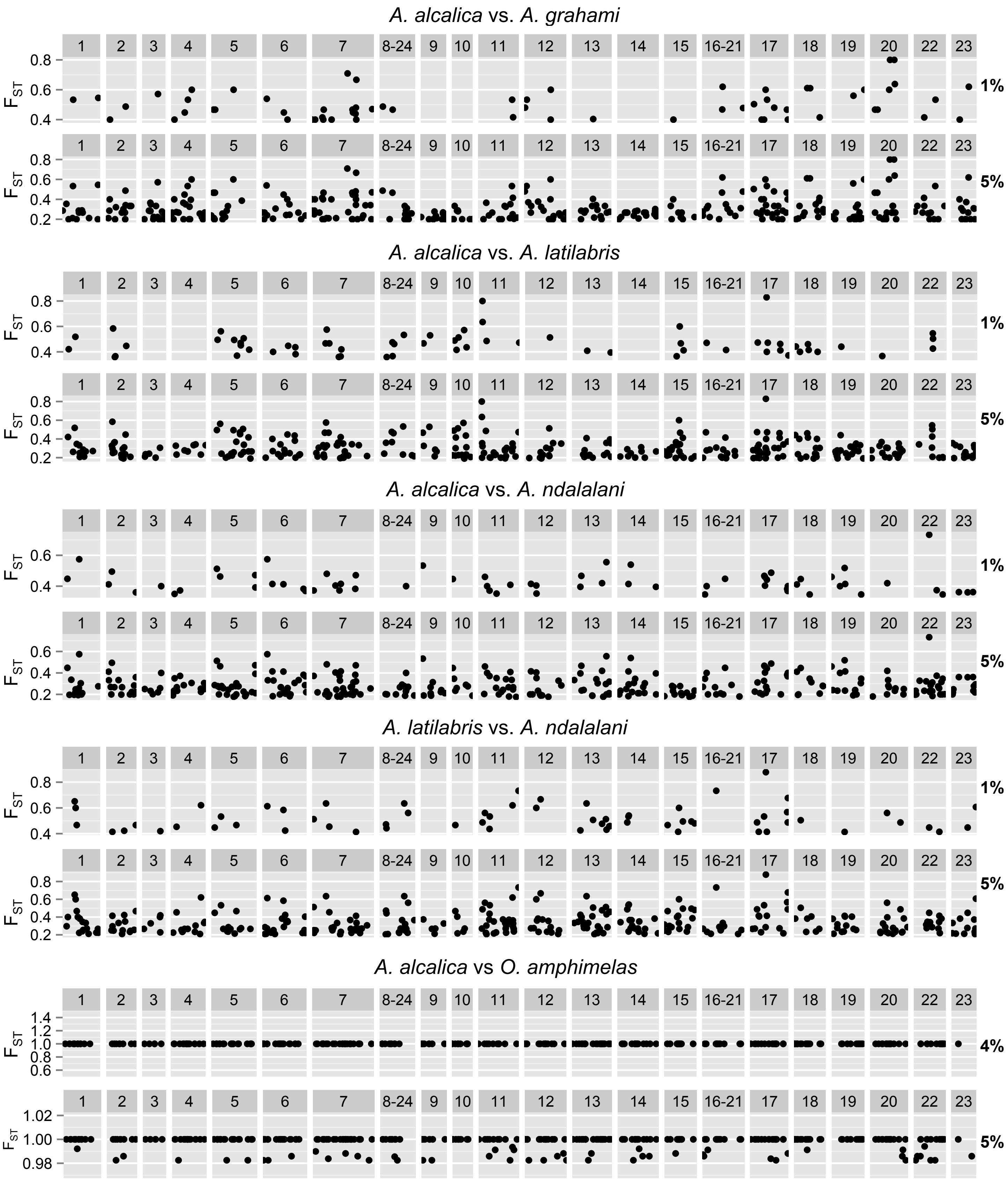

Figure S10. Plots of the highest scoring (top 1% and 5%) F_{ST} non-overlapping windows of 100kb. The highest-scoring windows in the outgroup (*O. amphimelas*) comparison all exhibited maximal values (F_{ST}=1.00), so all windows are shown at this value (equivalent to 4% of windows) rather than 1%. For the top 5% scoring windows, within-*Alcolapia* comparisons ranged from F_{ST}=0.2-0.8, while in the *O. amphimelas* comparison scores were considerably more constrained, ranging only between F_{ST}=0.98-1.00.

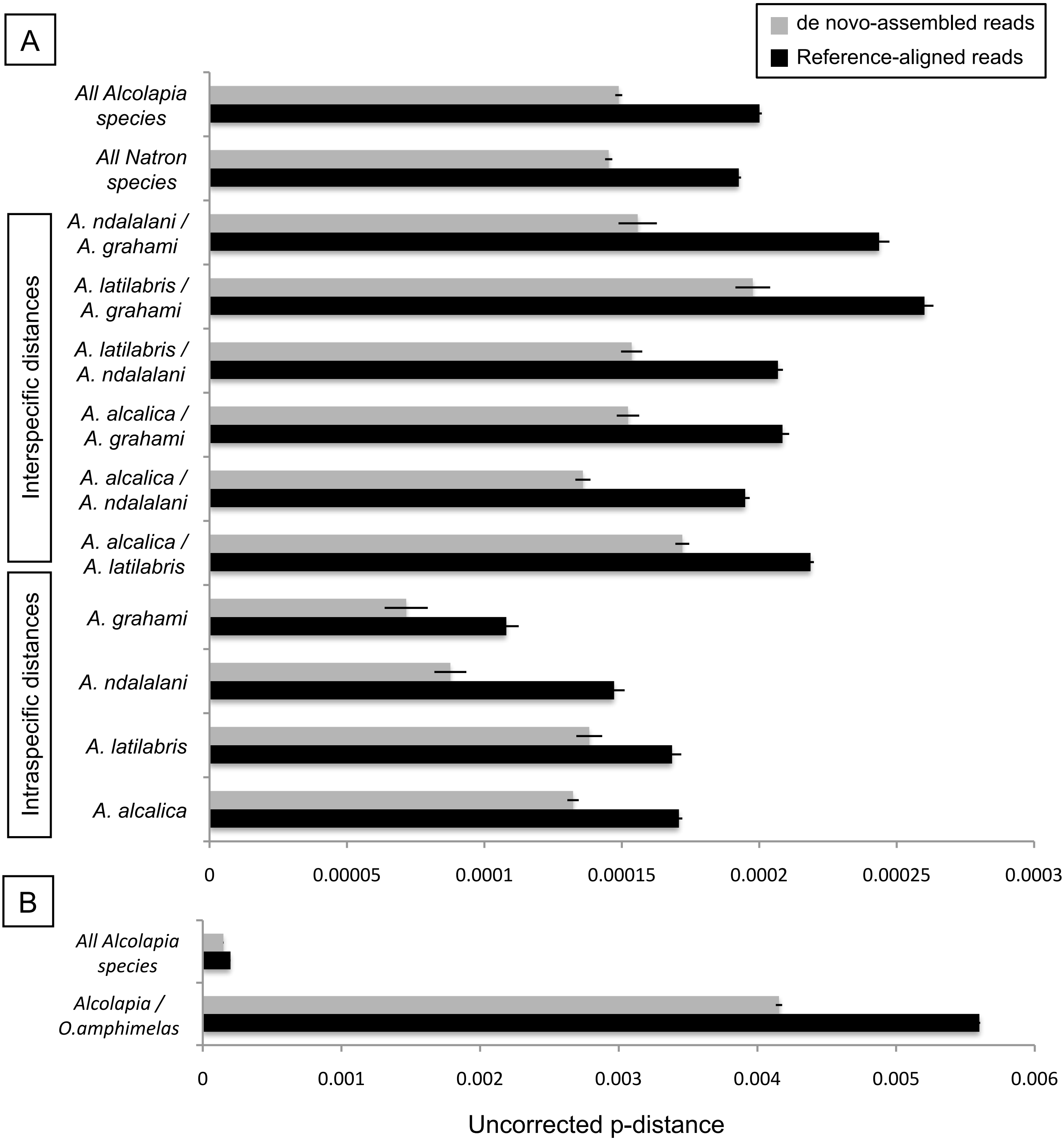

Figure S11. Mean inter-specimen uncorrected p-distance.
Comparison of filtered RAD data from aligned and de novo-assembled reads. A) Comparisons within *Alcolapia*. B) For comparison, the distance to the outgroup (*O. amphimelas*). Different scale axes are used for each graph (the first entry in each graph is for the same comparison, but at different scales). Error bars are +/- SEM.

SI Tables. All tables are also provided in editable (Excel) format, available in a separate file in the online version of this article.

Table S1. Collection co-ordinates and sequencing statistics per sample.

Samples in grey were excluded from downstream analyses due to poor quality and low total calls in the filtered dataset. OA: *O. amphimelas*; AA: *A. alcalica*; AB: *A. alcalica* blue morph; AY: *A. alcalica* yellow morph; AU: *A. alcalica* upturned-mouth morph; AL: *A. latilabris*; AN: *A. ndalalani*; AG: *A. grahami*.

	Sample ID	Accession reference ID	Sampling GPS coordinates	Sequenced reads (n)	Duplicates (n)	Duplicates (%)	Mapped (%)	Paired (%)	Total calls (n)	QC-filtered calls (n)	QC-filtered calls (%)	Mean mapping rate
O. amphimelas	002-001-OA	ERS652626	S -3.423333 E 35.851806	1357822	365705	26.93	89.73	81.15	19225625	N/A	N/A	*O. amphimelas* 89.85
	002-003-OA	ERS652628	S -3.423333 E 35.851806	3760790	1122956	29.86	89.67	80.88	27501927	12351820	44.91	
	002-004-OA	ERS652623	S -3.423333 E 35.851806	8688110	3077792	35.43	89.73	80.78	32790200	18166870	55.40	
	002-005-OA	ERS652624	S -3.423333 E 35.851806	5593242	1797032	32.13	89.72	80.95	28857245	15351777	53.20	
	004-029-OA	ERS652650	S -3.425306 E 35.343723	8499968	2816835	33.14	89.85	80.55	34897625	18049330	51.72	
	004-030-OA	ERS652670	S -3.425306 E 35.343723	4614448	1828295	39.62	89.85	81.38	25410823	12624979	49.68	
	004-032-OA	ERS652669	S -3.425306 E 35.343723	6429908	2044339	31.79	90.12	81.22	29516848	16229295	54.98	
	004-035-OA	ERS652671	S -3.425306 E 35.343723	6050738	1933421	31.95	90.16	81.46	30160099	16031277	53.15	
A. alcalica	005-160-AA	ERS652662	S -2.597583 E 35.918417	7962738	3157400	39.65	89.47	78.23	28647369	16339639	57.04	*A. alcalica* 88.79
	005-163-AA	ERS652663	S -2.597583 E 35.918417	12639506	5344313	42.28	89.40	78.24	31790644	19339959	60.84	
	005-164-AA	ERS652709	S -2.597583 E 35.918417	7767920	3151245	40.57	89.54	78.38	28511977	16007180	56.14	
	005-165-AA	ERS652665	S -2.597583 E 35.918417	6919164	2706673	39.12	89.42	78.62	28611615	15460588	54.04	
	006-058-AA	ERS652659	S -2.430397 E 35.895392	9480270	3404504	35.91	89.87	79.22	29482851	18084493	61.34	
	006-059-AA	ERS652658	S -2.430397 E 35.895392	8037504	2801660	34.86	89.62	78.93	29306108	17263844	58.91	
	006-073-AA	ERS652686	S -2.430397 E 35.895392	7386516	2595984	35.14	89.77	78.90	29135230	16782296	57.60	
	006-076-AA	ERS652684	S -2.430397 E 35.895392	8719750	3142721	36.04	89.75	78.87	29901941	17808281	59.56	
	009-230-AA	ERS652618	S -2.471278 E 35.887889	1627594	572245	35.16	89.42	78.94	19084112	4587454	24.04	
	009-231-AA	ERS652619	S -2.471278 E 35.887889	4312168	1629393	37.79	89.45	78.84	25365205	11775955	46.43	
	009-234-AA	ERS652617	S -2.471278 E 35.887889	10127960	4226515	41.73	89.31	78.18	31697682	17881771	56.41	
	009-243-AA	ERS652625	S -2.471278 E 35.887889	9616338	3959289	41.17	89.20	78.21	29774635	17480225	58.71	
	11A-320-AB	ERS652657	S -2.591000 E 36.009445	2475984	970742	39.21	89.87	79.55	21348756	7033004	32.94	
	11A-321-AB	ERS652656	S -2.591000 E 36.009445	6274036	2630185	41.92	89.74	79.41	26350715	14488129	54.98	
	11A-322-AB	ERS652655	S -2.591000 E 36.009445	9687160	4304246	44.43	89.77	79.23	28644508	17112417	59.74	
	11A-323-AY	ERS652654	S -2.591000 E 36.009445	11694544	5231031	44.73	89.82	79.34	29542463	18514362	62.67	
	11A-324-AY	ERS652653	S -2.591000 E 36.009445	8685264	3844753	44.27	89.82	79.44	27323911	16633029	60.87	
	11A-325-AY	ERS652652	S -2.591000 E 36.009445	9991060	4298967	43.03	89.57	78.88	27581617	17649780	63.99	
	11A-326-AY	ERS652651	S -2.591000 E 36.009445	10447384	4549111	43.54	89.61	79.07	28316443	17775105	62.77	
	11A-339-AB	ERS652672	S -2.591000 E 36.009445	10093142	4534603	44.93	89.74	79.02	27169435	17340274	63.82	
	012-517-AA	ERS652673	S -2.618972 E 35.999806	2150132	1409805	65.57	87.53	75.95	14321762	1203496	8.40	
	012-518-AA	ERS652674	S -2.618972 E 35.999806	9564398	6392842	66.84	87.09	75.22	26391555	8397909	31.82	
	012-521-AA	ERS652638	S -2.618972 E 35.999806	1193110	772867	64.78	86.67	75.71	9998081	313244	3.13	
	012-526-AA	ERS652641	S -2.618972 E 35.999806	4300436	2864522	66.61	87.42	75.84	19390154	3343132	17.24	

	Sample ID	Accession reference ID	Sampling GPS coordinates	Sequenced reads (n)	Duplicates (n)	Duplicates (%)	Mapped (%)	Paired (%)	Total calls (n)	QC-filtered calls (n)	QC-filtered calls (%)	Mean mapping rate
A. alcalica	015-733-AU	ERS652635	S -2.433361 E 36.10175	6589102	4432160	67.27	87.55	76.23	23136938	5559416	24.03	*A. alcalica* 88.79
	015-734-AU	ERS652700	S -2.433361 E 36.10175	24249950	15684157	64.68	84.99	72.88	34712858	19067432	54.93	
	015-750-AU	ERS652664	S -2.433361 E 36.10175	5625700	3608042	64.13	86.92	75.56	22447628	5440646	24.24	
	015-758-AU	ERS652642	S -2.433361 E 36.10175	12987418	7517598	57.88	89.26	78.61	28077266	16124699	57.43	
	015-848-AA	ERS652630	S -2.433361 E 36.10175	8412214	4502353	53.52	88.83	78.30	26656841	16556078	62.11	
	015-849-AA	ERS652631	S -2.433361 E 36.10175	7886154	4430135	56.18	88.95	78.50	25690733	16016409	62.34	
	015-850-AA	ERS652644	S -2.433361 E 36.10175	6311730	3354039	53.14	88.88	78.46	24020411	14792394	61.58	
	015-851-AA	ERS652643	S -2.433361 E 36.10175	9803922	5116278	52.19	88.62	77.64	26692651	15371665	57.59	
	017-083-AA	ERS652697	S -2.456278 E 36.087806	7439702	2553925	34.33	89.64	78.43	29072971	13810736	47.50	
	017-844-AA	ERS652634	S -2.456278 E 36.087806	12395920	7039379	56.79	89.05	78.41	28085487	12560157	44.72	
	017-846-AA	ERS652632	S -2.456278 E 36.087806	10438592	5865487	56.19	89.20	78.48	27009981	11431905	42.32	
	017-847-AA	ERS652633	S -2.456278 E 36.087806	11246308	6332246	56.31	89.10	78.40	27327923	15068717	55.14	
	019-879-AA	ERS652680	S -2.145833 E 36.05575	27008098	17754321	65.74	87.20	76.41	31615471	18962739	59.98	
	019-883-AA	ERS652701	S -2.145833 E 36.05575	11096528	7197186	64.86	87.86	76.91	24736152	12254593	49.54	
	019-887-AA	ERS652702	S -2.145833 E 36.05575	4305954	2473368	57.44	88.30	77.71	21038958	7229892	34.36	
	019-888-AA	ERS652703	S -2.145833 E 36.05575	8926152	5281875	59.17	86.54	75.89	25180896	12213631	48.50	
A. latilabris	005-086-AL	ERS652705	S -2.597583 E 35.918417	5299794	1723022	32.51	89.72	78.99	26629605	14388660	54.03	*A. latilabris* 88.74
	005-087-AL	ERS652704	S -2.597583 E 35.918417	7195264	2417648	33.60	89.59	78.77	28951967	16735599	57.80	
	005-088-AL	ERS652706	S -2.597583 E 35.918417	9458188	3352199	35.44	89.58	78.76	29921568	18547005	61.99	
	005-112-AL	ERS652647	S -2.597583 E 35.918417	4150534	1552605	37.41	89.32	78.49	24748665	11398690	46.06	
	009-247-AL	ERS652627	S -2.471278 E 35.887889	11118486	4640657	41.74	89.54	78.28	31325719	18615031	59.42	
	009-248-AL	ERS652621	S -2.471278 E 35.887889	12354302	5261344	42.59	89.57	78.42	31524470	19376823	61.47	
	009-249-AL	ERS652620	S -2.471278 E 35.887889	8642950	3539857	40.96	89.59	78.48	29261691	16895633	57.74	
	009-260-AL	ERS652694	S -2.471278 E 35.887889	8318970	3321266	39.92	89.51	78.68	29051678	16707856	57.51	
	11B-408-AL	ERS652668	S -2.591667 E 36.012056	4279346	1763096	41.20	89.84	79.39	23323430	11318570	48.53	
	11B-409-AL	ERS652667	S -2.591667 E 36.012056	8910514	3846933	43.17	89.76	78.92	26603729	16650052	62.59	
	11B-419-AL	ERS652679	S -2.591667 E 36.012056	4204078	1722750	40.98	89.88	79.63	23804721	11273496	47.36	
	11B-424-AL	ERS652689	S -2.591667 E 36.012056	4836192	3148127	65.10	86.67	75.34	21110867	4204765	19.92	
	012-567-AL	ERS652681	S -2.618972 E 35.999806	1479758	947123	64.01	85.34	74.07	11342001	487321	4.30	
	012-568-AL	ERS652685	S -2.618972 E 35.999806	11460052	7544751	65.84	87.39	75.79	28122043	11200347	39.83	
	012-572-AL	ERS652695	S -2.618972 E 35.999806	5437930	3517713	64.69	86.63	75.16	22682601	4851003	21.39	
	012-583-AL	ERS652649	S -2.618972 E 35.999806	6071594	4054002	66.77	87.14	75.69	22961856	4980778	21.69	
	017-788-AL	ERS652640	S -2.456278 E 36.087806	20111250	11830129	58.82	89.05	78.20	30313472	19209844	63.37	
	017-789-AL	ERS652639	S -2.456278 E 36.087806	17481210	10205842	58.38	89.15	78.40	29486809	18331005	62.17	
	017-813-AL	ERS652688	S -2.456278 E 36.087806	11007076	6154940	55.92	88.95	78.27	28032672	15308624	54.61	
	017-815-AL	ERS652687	S -2.456278 E 36.087806	6506730	3397642	52.22	88.59	77.86	25160380	11882911	47.23	

	Sample ID	Accession reference ID	Sampling GPS coordinates	Sequenced reads (n)	Duplicates (n)	Duplicates (%)	Mapped (%)	Paired (%)	Total calls (n)	QC-filtered calls (n)	QC-filtered calls (%)	Mean mapping rate
A. ndalalani	005-047-AN	ERS652660	S -2.597583 E 35.918417	3370746	1030405	30.57	89.68	78.90	24549929	10930759	44.52	*A. ndalalani* 88.75
	005-125-AN	ERS652710	S -2.597583 E 35.918417	9426726	3863169	40.98	89.21	77.95	29691805	17517360	59.00	
	005-126-AN	ERS652711	S -2.597583 E 35.918417	6624882	2595957	39.18	89.37	78.15	27799292	14926950	53.70	
	005-132-AN	ERS652629	S -2.597583 E 35.918417	7227458	2866770	39.66	89.26	78.14	28531493	15518207	54.39	
	11A-293-AN	ERS652699	S -2.591000 E 36.009445	3920796	1574459	40.16	89.29	78.86	22827759	10935424	47.90	
	11A-296-AN	ERS652698	S -2.591000 E 36.009445	6773356	2775470	40.98	89.67	79.26	26283459	15225966	57.93	
	11A-301-AN	ERS652645	S -2.591000 E 36.009445	5475056	2199871	40.18	89.39	78.79	24466137	13467064	55.04	
	11A-308-AN	ERS652616	S -2.591000 E 36.009445	1739654	676957	38.91	89.92	79.92	18722285	4795225	25.61	
	012-543-AN	ERS652707	S -2.618972 E 35.999806	5338238	3589974	67.25	87.37	75.91	20923070	4272347	20.42	
	012-545-AN	ERS652708	S -2.618972 E 35.999806	7347420	4936444	67.19	87.44	75.93	23581234	6377516	27.04	
	012-559-AN	ERS652622	S -2.618972 E 35.999806	5496640	3561925	64.80	87.30	75.83	22386833	5164868	23.07	
	012-562-AN	ERS652682	S -2.618972 E 35.999806	2752934	1745377	63.40	86.95	75.61	17204226	2166627	12.59	
	017-828-AN	ERS652661	S -2.456278 E 36.087806	15256986	8462298	55.47	88.72	77.68	29291995	17849726	60.94	
	017-830-AN	ERS652677	S -2.456278 E 36.087806	3343728	1756241	52.52	88.95	78.57	20432579	6679634	32.69	
	017-833-AN	ERS652675	S -2.456278 E 36.087806	11002348	5598092	50.88	88.45	77.71	28055294	16269760	57.99	
	017-839-AN	ERS652678	S -2.456278 E 36.087806	16709740	9782809	58.55	89.09	78.19	29204312	17900731	61.29	
A. grahami	018-859-AG	ERS652693	S -2.001139 E 36.231972	11332104	7316137	64.56	82.42	72.31	23007864	10787850	46.89	*A. grahami* 86.70
	018-869-AG	ERS652696	S -2.001139 E 36.231972	4807842	3357909	69.84	88.68	77.91	18492529	4395507	23.77	
	018-871-AG	ERS652683	S -2.001139 E 36.231972	38009624	25200827	66.30	87.96	77.04	33738637	21316544	63.18	
	018-900-AG	ERS652636	S -2.001139 E 36.231972	7910266	5012316	63.36	88.45	77.57	23267126	10405216	44.72	
	021-898-AG	ERS652692	S -1.844444 E 36.22425	2163910	1241469	57.37	87.69	77.22	16588390	2926140	17.64	
	021-899-AG	ERS652691	S -1.844444 E 36.22425	37937308	27520245	72.54	88.14	76.64	29148312	18680203	64.09	
	021-905-AG	ERS652637	S -1.844444 E 36.22425	22813336	14797603	64.86	87.29	76.42	28182867	17694293	62.78	
	021-910-AG	ERS652646	S -1.844444 E 36.22425	4502606	2915439	64.75	88.26	77.65	19456528	5554263	28.55	
	024-950-AG*	ERS652676	S -0.396028 E 36.107583	8081388	4910222	60.76	87.56	76.93	24052205	11267314	46.85	
	024-952-AG*	ERS652690	S -0.396028 E 36.107583	3456626	1932130	55.90	87.36	76.91	20057343	5936464	29.60	
	024-969-AG*	ERS652648	S -0.396028 E 36.107583	4365002	2897370	66.38	85.52	74.94	18065966	3914662	21.67	
	024-970-AG*	ERS652666	S -0.396028 E 36.107583	9962378	4713690	47.31	84.30	74.34	26614209	15712608	59.04	
	**TOTAL:**			836345864	439397761	N/A	N/A	N/A	N/A	N/A	N/A	N/A
	**MEAN:**			8711936	4577060	49.75	88.64	77.94	25838916	12850898	47.10	N/A

*indicates sample collected from introduced population at Lake Nakuru.
Voucher specimens (unaccessioned) are held in the Day lab at UCL.

Table S2. Data subsets and respective analyses conducted on RAD data

	Description	Sequences	Sites (n)	Variant (%)	Analyses	Figure
**A**	Full alignment of all mapped, filtered sites with data for >1 individual	n=92 (ref. *O. niloticus*, 7 *O. amphimelas*, 84 *Alcolapia*)	28,560,698	1.91	p-distance	SI 11
**B**	Full alignment of all mapped, quality filtered sites, reduced taxa	n=25 (ref. *O. niloticus*, 4 *O. amphimelas*, 20 *Alcolapia*)	26,135,098	1.71	RAxML GTRGAMMA model	2A,B
**C**	Full alignment excluding reads not mapped to assigned linkage groups	n=92 (ref. *O. niloticus*, 7 *O. amphimelas*, 84 *Alcolapia*)	22,249,264	1.82	LD, f_4 F_{ST} (EggLib method), Sliding-window F_{ST}	4, Table1 SI 2,
**D**	Variable sites (SNPs) across full alignment	n=92 (ref. *O. niloticus*, 7 *O. amphimelas*, 84 *Alcolapia*)	544,916	100	RAxML GTRGAMMA model	2C
**E**	SNPs across full alignment, excluding ambiguous bases	n=92 (ref. *O. niloticus*, 7 *O. amphimelas*, 84 *Alcolapia*)	366,016	100	RAxML GTRGAMMA model, corrected for ascertainment bias	SI 3
**F**	Biallelic unlinked SNPs 12 populations (n=4 per population), no missing data	n=48 (38 *A. alcalica*, 12 *A. grahami*, 19 *A. latilabris*, 15 *A. ndalalani*)	1,266	100	SNAPP	SI 5
**G**	Full alignment, de novo assembled, filtered sites	n=91 (7 *O. amphimelas*, 84 *Alcolapia*)	436,839	1.33	RAxML GTRGAMMA model	SI 4A
**H**	SNPs from de novo assembled alignment	n=91 (7 *O. amphimelas*, 84 *Alcolapia*)	5,832	100	RAxML GTRGAMMA model	SI 4B
**J**	SNPs from de novo, no ambiguous bases	n=91 (7 *O. amphimelas*, 84 *Alcolapia*)	1,898	100	RAxML GTRGAMMA model, _ASC model	SI 4C
**K**	Combined mapped and de novo reads, SNPs	n=91 (7 *O. amphimelas*, 84 *Alcolapia*)	550,748	100	RAxML GTRGAMMA model	SI 4D
**L**	Combined mapped and de novo reads, SNPs, no ambiguous bases	n=91 (7 *O. amphimelas*, 84 *Alcolapia*)	182,909	100	RAxML GTRGAMMA model, _ASC model	SI 4E
**M**	Biallelic SNPs across *Alcolapia*	n=84 (38 *A. alcalica*, 12 *A. grahami*, 19 *A. latilabris*, 15 *A. ndalalani*)	246,336	100	STRUCTURE, SplitsTree	2D, SI 6
**N**	Biallelic unlinked SNPs across *Alcolapia*	n=84 (38 *A. alcalica*, 12 *A. grahami*, 19 *A. latilabris*, 15 *A. ndalalani*)	2,297	100	STRUCTURE	3, SI 6
**P**	*A. alcalica* biallelic SNPs	n=38 *A. alcalica*	173,964	100	STRUCTURE	SI 8
**Q**	*A. alcalica* unlinked SNPs	n=38 *A. alcalica*	2,222	100	STRUCTURE	SI 8
**R**	Biallelic SNPs, max missing data 10%	n=84 (38 *A. alcalica*, 12 *A. grahami*, 19 *A. latilabris*, 15 *A. ndalalani*)	31,555	100	F_{ST} (Arlequin)	Table SI 3
**S**	Biallelic SNPs, MAF >0.1, max. missing data 25%	n=16 (8 *A. alcalica*, 8 *A. grahami*)	23,264	100	Bayescan	Table 2
**T**	Biallelic SNPs, MAF >0.1, max. missing data 25%	n=16 (8 *A. alcalica*, 8 *A. latilabris*)	30,841	100	Bayescan	Table 2
**U**	Biallelic SNPs, MAF >0.1, max. missing data 25%	n=16 (8 *A. alcalica*, 8 *A. ndalalani*)	28,026	100	Bayescan	Table 2
**V**	Biallelic SNPs, MAF >0.1, max. missing data 25%	n=16 (8 *A. latilabris*, 8 *A. ndalalani*)	22,946	100	Bayescan	Table 2
**W**	Biallelic SNPs, MAF >0.1, max. missing data 25%	n=14 (7 *A. alcalica*, 7 *O. amphimelas*)	82,474	100	Bayescan	Table 2
**X**	Biallelic unlinked SNPs, sympatric *Alcolapia*	n=24 (8 *A. alcalica*, 8 *A. latilabris*, 8 *A. ndalalani*)	2,227	100	STRUCTURE	SI 7

Table S3. Population pairwise F_{ST}.

Below diagonal: maximum level missing data 10%, 31,555 SNPs (dataset R), F_{ST} calculated in Arlequin accounting for sample size.

		A. alcalica									*A. latilabris*					*A. ndalalani*				*A. grahami*		
	**Site:**	**005**	**006**	**009**	**011**	**012**	**015**	**015***	**017**	**019**	**005**	**009**	**011**	**012**	**017**	**005**	**11A**	**012**	**017**	**018**	**021**	**024**
A. alcalica	005	-																				
	006	0.075	-																			
	009	0.038	0.039	-																		
	011	0.049	0.058	0.011	-																	
	012	0.056	0.084	0.017	0.016	-																
	015	0.099	0.078	0.068	0.074	0.113	-															
	015*	0.072	0.058	0.044	0.056	0.083	0.001	-														
	017	0.029	0.053	-0.003	0.001	0.004	0.074	0.034	-													
	019	0.042	0.027	0.001	0.020	0.038	0.057	0.038	0.022	-												
A. latilabris	005	0.063	0.117	0.072	0.083	0.096	0.126	0.105	0.059	0.087	-											
	009	0.146	0.158	0.085	0.127	0.147	0.174	0.151	0.135	0.116	0.132	-										
	011	0.102	0.124	0.078	0.084	0.104	0.138	0.115	0.075	0.091	0.080	0.082	-									
	012	0.132	0.153	0.101	0.115	0.118	0.176	0.153	0.110	0.121	0.106	0.146	0.026	-								
	017	0.053	0.137	0.042	0.043	0.050	0.100	0.072	0.018	0.039	0.067	0.093	0.033	0.030	-							
A. ndalalani	005	0.090	0.126	0.071	0.076	0.109	0.154	0.120	0.069	0.081	0.108	0.191	0.140	0.159	0.099	-						
	011	0.113	0.137	0.077	0.062	0.072	0.157	0.134	0.068	0.089	0.130	0.163	0.119	0.151	0.075	0.122	-					
	012	0.072	0.101	0.047	0.031	0.065	0.126	0.105	0.037	0.058	0.105	0.150	0.088	0.116	0.041	0.092	-0.014	-				
	017	0.101	0.119	0.076	0.091	0.119	0.136	0.107	0.065	0.089	0.126	0.203	0.141	0.152	0.063	0.143	0.095	0.092	-			
A. grahami	018	0.112	0.123	0.100	0.100	0.119	0.137	0.114	0.075	0.089	0.150	0.185	0.154	0.189	0.108	0.164	0.168	0.141	0.156	-		
	021	0.088	0.103	0.077	0.090	0.093	0.119	0.094	0.056	0.070	0.126	0.170	0.140	0.170	0.097	0.124	0.150	0.123	0.139	-0.010	-	
	024	0.108	0.109	0.088	0.106	0.115	0.135	0.112	0.068	0.079	0.137	0.172	0.147	0.179	0.101	0.154	0.164	0.132	0.142	0.014	-0.007	-

*indicates *A. alcalica* upturned-mouth morph from site 015. Site 011 *A. alcalica* includes comparisons for the blue morph only (n=4). Site 017 *A. alcalica* population excludes the two samples designated *A.* aff. *ndalalani* from the ML analysis.

None of the comparisons was significant after Bonferroni correction for multiple testing (α=0.0002).

	<0.03		<0.09		<0.15		>0.18
	<0.06		<0.12		<0.18		

Table S4. Bayescan outlier loci for *A. alcalica* vs *A. grahami* comparison.

Chromosome	Position (bp)	q-value	alpha	F_{ST}
LG1	4880146	0.0586	1.4117	0.3375
LG2	12745226	0.0013	1.9795	0.4525
LG3	1061977	0.0101	1.5942	0.3687
LG3	3742179	0.0502	1.3959	0.3340
LG4	26817200	0.0004	2.0652	0.4712
LG5	3793947	0.0559	1.3943	0.3338
LG7	29281646	0.0056	1.6455	0.3785
LG7	36442432	0.0471	1.3312	0.3194
LG8-24	12272405	0.0797	1.1819	0.2964
LG8-24	12272651	0.0438	1.3566	0.3242
LG8-24	25594923	0.0137	1.5359	0.3562
LG8-24	25594939	0.0171	1.5360	0.3567
LG11	3550520	0.0041	1.6499	0.3798
LG11	18919683	0.0019	1.9887	0.4548
LG11	18919756	0.0007	2.0520	0.4679
LG11	18924298	0.0021	1.9969	0.4560
LG11	19069888	0.0083	1.5753	0.3633
LG11	27805595	0.0024	1.9866	0.4538
LG11	28559170	0.0376	1.3798	0.3284
**LG11**	**28627402**	**0.0194**	**1.5180**	**0.3538**
LG12	11467527	0.0639	1.3757	0.3308
LG12	17142862	0.0665	1.3650	0.3285
LG12	31972729	0.0155	1.5884	0.3681
LG16-21	16552748	0.0923	1.1154	0.2828
LG16-21	27504075	0.0840	1.1515	0.2901
LG16-21	30216099	0.0531	1.3876	0.3326
LG16-21	34046293	0.0964	1.0475	0.2689
LG16-21	34070668	0.0753	1.1640	0.2922
LG17	8127213	0.0005	2.1170	0.4827
LG17	11752066	0.0407	1.3877	0.3306
LG18	25974009	0.0708	1.2016	0.2995
LG20	15632809	0.0002	2.1519	0.4904
LG20	15633007	0.0007	2.0637	0.4707
LG20	16552727	0.0344	1.3964	0.3306
LG20	20086617	0.0267	1.4571	0.3418
LG23	1333779	0.0613	1.3773	0.3307
UNK1	862049	0.0010	2.0539	0.4689
UNK2	924286	0.0219	1.4948	0.3498
UNK37	1009096	0.0882	1.1253	0.2850
UNK73	339327	0.0317	1.3985	0.3302
UNK107	485448	0.0290	1.4624	0.3433
UNK121	459348	0.0070	1.6592	0.3818
UNK121	526329	0.0016	2.0066	0.4587
UNK268	158116	0.0118	1.5402	0.3570
UNK495	49297	0.0243	1.3599	0.3199

Yellow shading indicates loci identified as outliers at FDR=0.05; green indicates additional outlier loci identified at FDR=0.10. LG refers to linkage groups from reference genome used for alignment (*O. niloticus*), UNK refer to unplaced scaffolds within the genome. Rows in bold indicate SNPs that were identified as outliers in multiple species comparisons.

Table S5. Bayescan outlier loci for A. alcalica vs A. latilabris comparison.

Chromosome	Position (bp)	q-value	alpha	F_{ST}
LG1	4417090	0.0279	1.7238	0.1941
LG1	5534111	0.0454	1.6156	0.1827
**LG1**	**9901931**	**0.0613**	**1.4219**	**0.1569**
LG1	10030509	0.0000	2.6671	0.3491
LG2	16182727	0.0302	1.5967	0.1750
**LG4**	**6651728**	**0.0863**	**1.3118**	**0.1502**
LG5	4507212	0.0001	2.2695	0.2731
LG5	7280901	0.0002	2.2676	0.2726
LG5	16192393	0.0010	2.2432	0.2706
LG5	18234447	0.0964	1.4024	0.1683
**LG5**	**26185535**	**0.0018**	**1.9564**	**0.2204**
LG6	8236011	0.0208	1.8866	0.2177
LG7	11499980	0.0112	1.7708	0.1970
LG7	19014506	0.0531	1.5305	0.1732
LG7	20824106	0.0669	1.5258	0.1756
LG7	22963036	0.0176	1.7324	0.1905
LG7	22963066	0.0793	1.3838	0.1611
LG11	2931392	0.0014	1.9671	0.2235
LG11	6384781	0.0929	1.3452	0.1574
**LG11**	**28627402**	**0.0586**	**1.4238**	**0.1569**
LG12	21251407	0.0722	1.4556	0.1655
LG13	28499021	0.0503	1.7080	0.1989
LG15	9225207	0.0403	1.4965	0.1635
LG16-21	3406817	0.0241	1.8808	0.2173
**LG17**	**12926209**	**0.0559**	**1.5960**	**0.1842**
**LG17**	**13007816**	**0.0008**	**2.0367**	**0.2327**
**LG17**	**13007817**	**0.0004**	**2.0197**	**0.2299**
**LG17**	**13087966**	**0.0003**	**2.0369**	**0.2321**
**LG17**	**13091875**	**0.0006**	**2.0384**	**0.2327**
LG17	13270025	0.0024	2.0738	0.2419
LG17	20107222	0.0259	1.6703	0.1836
**LG17**	**24302170**	**0.0002**	**2.1499**	**0.2514**
LG17	24496689	0.0896	1.3553	0.1571
LG18	18845531	0.0192	1.7296	0.1903
**LG19**	**7542228**	**0.0829**	**1.3681**	**0.1599**
LG22	15261172	0.0641	1.4670	0.1658
LG22	15287396	0.0033	1.9595	0.2231
LG22	15287743	0.0479	1.6027	0.1819
LG22	15308783	0.0159	1.7619	0.1956
LG22	24241771	0.0050	1.9478	0.2221
UNK5	717734	0.0159	1.8481	0.2093
UNK24	342964	0.0075	1.7982	0.1976
UNK38	128450	0.0695	1.4637	0.1662
UNK43	367041	0.0059	1.8117	0.1995
UNK50	526645	0.0327	1.5769	0.1735
UNK50	820921	0.0067	1.8691	0.2082
UNK56	765711	0.0757	1.3194	0.1496
UNK89	110278	0.0428	1.7166	0.1987
UNK95	37233	0.0000	2.3780	0.2906
UNK103	307429	0.0352	1.6416	0.1842
UNK168	164402	0.0095	1.8605	0.2107
UNK168	356741	0.0129	1.8479	0.2091
UNK181	262893	0.0041	2.0368	0.2363
UNK234	123146	0.0224	1.8909	0.2175
UNK300	208694	0.0376	1.6563	0.1864

Table S6. Bayescan outlier loci for *A. alcalica* vs *A. ndalalani* comparison.

Chromosome	Position (bp)	q-value	alpha	F_{ST}
LG1	3698842	0.0352	1.7403	0.1900
**LG1**	**9194783**	**0.0163**	**1.9405**	**0.2195**
LG1	11666374	0.0633	1.6592	0.1895
LG1	11666684	0.0329	1.8688	0.2112
LG1	13686376	0.0484	1.7399	0.1944
LG1	13779141	0.0862	1.5656	0.1792
LG1	25525499	0.0458	1.6611	0.1811
LG2	2120367	0.0404	1.6649	0.1809
LG2	4290091	0.0684	1.5324	0.1724
LG2	7609602	0.0935	1.5470	0.1769
**LG5**	**26185535**	**0.0004**	**2.3241**	**0.2798**
**LG6**	**4117668**	**0.0509**	**1.7374**	**0.1941**
**LG6**	**17839834**	**0.0823**	**1.5724**	**0.1796**
LG6	18215001	0.0588	1.7313	0.1962
LG7	22543193	0.0192	2.0224	0.2336
LG7	30162091	0.0780	1.5133	0.1698
**LG8-24**	**24244837**	**0.0371**	**1.8483**	**0.2084**
**LG8-24**	**24244838**	**0.0279**	**1.8670**	**0.2107**
**LG17**	**12997082**	**0.0109**	**2.1182**	**0.2495**
LG18	5413311	0.0973	1.4507	0.1629
LG19	10882365	0.0560	1.5684	0.1697
LG22	12765765	0.0240	1.7900	0.1981
UNK33	1062913	0.0532	1.7395	0.1953
UNK38	608444	0.0899	1.4973	0.1688
UNK38	705388	0.0307	1.7390	0.1895
UNK38	1274353	0.0432	1.6733	0.1820
UNK86	245483	0.0780	1.5065	0.1689

Table S7. Bayescan outlier loci for *A. latilabris* vs *A. ndalalani* comparison.

Chromosome	Position (bp)	q-value	alpha	F_{ST}
**LG1**	**9194783**	**0.0093**	**1.8032**	**0.2771**
**LG1**	**9901931**	**0.0126**	**1.5955**	**0.2388**
LG2	9934672	0.0660	1.3796	0.2180
LG2	14429342	0.0310	1.5541	0.2389
**LG4**	**6651728**	**0.0078**	**1.7755**	**0.2716**
LG4	24754695	0.0777	1.2336	0.1927
LG4	24754717	0.0865	1.1954	0.1872
LG4	24754718	0.0754	1.2382	0.1939
LG4	24754744	0.0886	1.2212	0.1920
LG5	15313237	0.0109	1.7306	0.2633
LG5	16192182	0.0197	1.6607	0.2536
LG5	28714028	0.0012	1.9178	0.2966
LG5	30767236	0.0430	1.4680	0.2278
**LG6**	**4117668**	**0.0156**	**1.6580**	**0.2514**
LG6	14339857	0.0377	1.4534	0.2200
**LG6**	**17839834**	**0.0027**	**1.8484**	**0.2838**
LG6	20814433	0.0327	1.5366	0.2361
LG7	12555135	0.0000	2.4125	0.3987
LG7	19277166	0.0256	1.4870	0.2239
LG8-24	11575335	0.0550	1.4694	0.2308
LG8-24	11575814	0.0101	1.7739	0.2717
**LG8-24**	**24244837**	**0.0006**	**1.9295**	**0.2986**
**LG8-24**	**24244838**	**0.0021**	**1.8577**	**0.2857**
LG9	7012601	0.0185	1.6851	0.2577
LG10	9537712	0.0050	1.7580	0.2680
LG11	3792961	0.0978	1.1719	0.1873
LG11	33302772	0.0135	1.6751	0.2546
LG11	33302799	0.0175	1.6483	0.2500
LG11	33303048	0.0931	1.1872	0.1893
LG12	13411277	0.0593	1.3806	0.2155
LG12	13411307	0.0614	1.3866	0.2170
LG12	13411469	0.0572	1.3925	0.2174
LG12	20337529	0.0410	1.4379	0.2185
LG13	16810867	0.0033	1.8204	0.2792
LG13	16811174	0.0450	1.4752	0.2287
LG13	17422310	0.0906	1.2132	0.1905
LG13	24877405	0.0707	1.2457	0.1939
LG15	9632346	0.0754	1.2329	0.1929
LG16-21	3603414	0.0069	1.7480	0.2664
LG16-21	3636514	0.0509	1.5124	0.2372
LG16-21	11594518	0.0166	1.6668	0.2539
LG16-21	19134767	0.0489	1.4723	0.2285

Table continues on next page.

Table S7. Bayescan outlier loci for *A. latilabris* vs *A. ndalalani* comparison (cont).

Chromosome	Position (bp)	q-value	alpha	F_{ST}
LG17	7574728	0.0117	1.7670	0.2712
**LG17**	**12926209**	**0.0000**	**2.5280**	**0.4228**
**LG17**	**12997082**	**0.0001**	**2.3101**	**0.3776**
**LG17**	**13007816**	**0.0000**	**2.1734**	**0.3470**
**LG17**	**13007817**	**0.0000**	**2.1779**	**0.3472**
**LG17**	**13087966**	**0.0001**	**2.1848**	**0.3488**
**LG17**	**13091875**	**0.0001**	**2.1285**	**0.3372**
**LG17**	**24302170**	**0.0344**	**1.4480**	**0.2192**
LG17	31257285	0.0156	1.6701	0.2547
LG18	5413298	0.0821	1.2147	0.1903
LG18	18845531	0.0007	2.0042	0.3139
LG19	3811871	0.0223	1.6530	0.2542
**LG19**	**7542228**	**0.0041**	**1.8116**	**0.2771**
LG19	7542249	0.0239	1.6586	0.2565
LG19	7544806	0.0209	1.6658	0.2545
LG19	7545050	0.0394	1.5504	0.2396
LG19	10903764	0.0361	1.5597	0.2408
LG19	18829834	0.0955	1.2428	0.1999
LG20	24059412	0.0060	1.7615	0.2691
LG23	20226647	0.0843	1.2103	0.1898
UNK1	878607	0.0003	2.0653	0.3250
UNK1	1017116	0.0085	1.7675	0.2702
UNK5	749629	0.0016	1.9485	0.3035
UNK6	845852	0.0470	1.5323	0.2398
UNK37	54509	0.0293	1.4777	0.2237
UNK61	362040	0.0636	1.3645	0.2133
UNK73	530939	0.0009	2.0214	0.3173
UNK73	767643	0.0275	1.5579	0.2394
UNK86	245483	0.0528	1.4519	0.2258
UNK88	656959	0.0005	2.0535	0.3232
UNK89	378951	0.0684	1.3800	0.2189
UNK95	37233	0.0003	2.0702	0.3263

Table S8. Outlier SNPs and predicted gene annotations.
SNPs identified as F_{ST} outliers in more than one species comparison from Bayescan analyses and the corresponding predicted gene annotation from NCBI (*O. niloticus* annotation release 101). The final row includes the window identified as a peak in the D_{XY} sliding-window analysis, and includes all annotated genes within this 1.5MB region (although this peak was not identified in the Bayescan analysis).

**Chromosome: bp**	**Gene annotation**
LG1: 9901931	Synaptotagmin-7-like (LOC100697404)
LG1: 9194783	Growth arrest-specific protein 2-like (LOC100704610)
LG4: 6651728	Fascin-like (LOC100703702)
LG5: 26185535	No annotation
LG6: 4117668	Suppressor of cytokine signaling 7-like (LOC100711954)
LG6: 17839834	Glyoxylate reductase/hydroxypyruvate reductase-like (LOC100699147)
LG8-24: 24244837, 24244838	No annotation
LG11: 28627402	Potassium voltage-gated channel subfamily V member 1-like (LOC100693033)
LG17: 12926209	Exportin-T-like (LOC100695406)
LG17: 12997082, 13007816,13007817	No annotation
LG17: 13087966, 13091875	Parathyroid hormone-related protein-like (LOC100694874)
LG:17: 24302170	No annotation
LG19: 7542228	Mitochondrial 2-oxodicarboxylate carrier-like (LOC100696078)
LG23: 3500000-5000000	Probable G-protein coupled receptor 128-like (LOC102080577)<br>DPH3 homolog (LOC100702351)<br>Opsin-3-like (LOC100693866)<br>NACHT, LRR and PYD domains-containing protein 12-like (LOC100699918)<br>Leucine-rich repeat flightless-interacting protein 2-like (LOC100700729)<br>Ribonuclease inhibitor-like (LOC100702088)<br>Protein NLRC3-like<br>Tumor necrosis factor receptor superfamily member 14-like (LOC100703705)<br>Tumor necrosis factor receptor superfamily member 5-like (LOC102082670)<br>Caspase-1-like (LOC102076866)<br>Replication factor C subunit 4-like (LOC100704243)<br>Guanine nucleotide exchange factor DBS-like (LOC100704513)<br>Lactosylceramide 1,3-N-acetyl-beta-D-glucosaminyltransferase A-like (LOC102082918)<br>Epithelial cell transforming sequence 2 oncogene (ect2)<br>Neuroligin-1-like (LOC100705052)

Table S9. Mantel test results.
Mean genetic pairwise distance or population pairwise F_{ST} vs geographic distance between sampling sites.

	**Uncorrected p-distance**				**Pairwise F_{ST}**			
**Geographic distance:**	**Straight-line**		**Lake-perimeter**		**Straight-line**		**Lake-perimeter**	
	**Observation**	**P-value**	**Observation**	**P-value**	**Observation**	**P-value**	**Observation**	**P-value**
**Population comparisons**								
A. alcalica	-0.055	0.513	0.118	0.355	-0.027	0.398	0.013	0.456
A. latilabris	0.604	0.112	0.647	0.083	0.169	0.344	0.213	0.325
A. ndalalani	0.859	0.037	0.879	0.084	0.731	0.128	0.735	0.123
A. grahami	-0.092	0.502	-	-	0.583	0.337	-	-
